# Evaluation of the impact of ecological factors on the habitat suitability and bioactive components accumulation of the medicinal holoparasitic plant *Cynomorium songaricum* using machine learning models

**DOI:** 10.3389/fpls.2025.1586682

**Published:** 2025-07-17

**Authors:** Jiacheng Ji, Xinxin Wei, Huan Guan, Zikang Jin, Xin Yue, Zhuoran Jiang, Youla Su, Shuying Sun, Guilin Chen

**Affiliations:** ^1^ Key Laboratory of Herbage and Endemic Crop Biology, Ministry of Education, School of Life Sciences, Inner Mongolia University, Hohhot, China; ^2^ The Good Agriculture Practice Engineering Technology Research Center of Chinese and Mongolian Medicine in Inner Mongolia, Inner Mongolia University, Hohhot, China; ^3^ School of Pharmacy, Inner Mongolia Medical University, Hohhot, China

**Keywords:** *Cynomorium songaricum* Rupr., environmental factors, habitat suitability, machine learning models, bioactive components, high-quality growing zones

## Abstract

The efficacy of traditional Chinese medicine is determined by its bioactive components, which exhibit variability depending on environmental conditions and hereditary influences. In this study, we focus on *Cynomorium songaricum* Rupr., a medicinally significant species facing sustainability challenges. However, the ecological drivers governing its distribution, as well as the relationship between environmental factors and bioactive components, remain unclear. Thus, we sampled 28 representative distribution areas of *C. songaricum* in China. Employing Maximum Entropy (MaxEnt) modeling, we projected current and future (2050s-2090s) habitat suitability under four emission scenarios. Notably, species distribution exhibited expansion (8.03%-29.06% range increase across scenarios) with precipitation of the wettest month (BIO13) and soil pH emerging as key drivers (combined contribution >49%). Ultra-performance liquid chromatography (UPLC) fingerprinting combined with machine learning regression was applied to quantify six key bioactive components in *C. songaricum*, 3,4-dihydroxybenzaldehyde, catechin, epicatechin, ursolic acid, total phenolics, and crude polysaccharides—revealing significant concentration variations among geographically distinct populations. Slope gradient (slope), min temperature of coldest month (BIO6), precipitation of coldest quarter (BIO19), sunshine duration in growing season(hsdgs), and isothermality (BIO3) were identified as key regulatory factors influencing the accumulation of multiple components. Specifically, slope acted as a key shared negative regulator for 3,4-dihydroxybenzaldehyde, catechin, and crude polysaccharides. BIO6 served as a key shared positive regulator for catechin and total phenolics, while functioning as a key negative regulator for ursolic acid. BIO19 was identified as a key shared negative regulator for catechin and epicatechin. Hsdgs acted as a key positive regulator for ursolic acid while negatively regulating crude polysaccharides. Additionally, BIO3 served as a key shared positive regulator for both ursolic acid and total phenolics. This study provides the scientific basis for enabling targeted cultivation zones that balance therapeutic compound yield with arid ecosystem conservation.

## 1 Introduction

Climate change significantly impacts the distribution of various ecosystems, and the effects of future climate change will likely modify the habitat, scope, and distribution of myriad species (Li et al., 2022). According to the Intergovernmental Panel on Climate Change (IPCC) special report “Global Warming of 1.5 °C”, the average global surface temperature is anticipated to rise 1.5°C between 2030 and 2052 (Allen et al., 2018). Climate warming and increasingly extreme weather events (severe droughts, heavy rainfalls, heat waves, cold snaps) can significantly alter species habitats and phenology. These changes have triggered environmental challenges such as altered spatial patterns of species. Consequently, biodiversity and sustainable development are increasingly threatened (Kerr et al., 2007; Li et al., 2014, Li et al., 2019; Shen et al., 2021). Dryland ecosystems, which cover approximately 40% of the Earth’s terrestrial surface, play a critical role in the global carbon cycle (Wang et al., 2022c). Driven by both climate change and natural climate variability (e.g., El Niño/Southern Oscillation), global warming is expected to increase environmental variability, including fluctuations in precipitation, temperature, and soil conditions (Wang et al., 2022c). These combined stresses collectively intensify the impact of climate change on plant distribution in arid regions. Extreme drought events triggered by precipitation variability can initiate hydraulic failure in plants, leading to tissue-level hydraulic collapse and cytorrhysis in affected cells (McDowell et al., 2022). Meanwhile, drought-induced carbon starvation may compromise the energy supply required to sustain water transport, while also weakening plant defense against insects and/or pathogens, ultimately exacerbating xylem embolism (McDowell et al., 2022). Salinization induced by climate change is a common risk in arid regions, where salt accumulation in the rhizosphere may exceed plant tolerance thresholds, leading to osmotic stress and nutrient imbalance, ultimately limiting plant growth (Hassani et al., 2021).

Secondary metabolites play a key role in helping plants with diverse genetic backgrounds adapt to environmental changes and are therefore highly sensitive to climate fluctuations (Sun et al., 2023). In recent years, sustained interest in natural medicines and functional plant ingredients has sharply increased global demand for research and development of physiologically active secondary metabolites from medicinal plants. However, because the synthesis of these components remains technically challenging, medicinal plants continue to be the primary source of such bioactive components (Sun et al., 2023). Their responses to environmental factors are highly variable: elevated CO_2_ concentrations and higher temperatures generally enhance phenolic compounds such as chlorogenic acid and rutin (Ghasemzadeh et al., 2012; Nguyen et al., 2019; Guo et al., 2020), yet decreases have also been reported (Chang et al., 2016; Jia et al., 2016). Simulated nitrogen deposition can promote plant growth and primary metabolism, but may suppress phenolic biosynthesis (Sun et al., 2020). Moreover, climate variables often exert non-linear effects; in tomatoes, temperature, relative humidity, and CO_2_ concentration all influence the photosynthetic rate. Notably, the highest photosynthetic rate was not observed during the period with the highest CO_2_ concentration (Chen et al., 2025; Devadze et al., 2025). Because the accumulation of these compounds is typically governed by multiple interacting ecological drivers, establishing quantitative relationships between environmental change and key pharmacologically active constituents remains challenging. Given that the overall therapeutic efficacy of medicinal plants relies on the synergistic actions of multiple components, a systematic approach is urgently needed to assess how environmental changes affect herbal quality.

The accelerated warming that has been observed in dryland regions over recent decades is expected to continue, with deserts projected to warm faster than many other terrestrial areas. Relative to the historical period (1961–1990), surface warming in drylands is projected to reach approximately 6.5°C under the high-emissions scenario (RCP8.5) and about 3.5°C under the low-to-moderate emissions scenario (RCP4.5) by the end of this century (Stringer et al., 2021). The ongoing rise in temperature is anticipated to further intensify drought stress and habitat degradation, posing dual challenges to the geographic distribution and medicinal quality of medicinal plants. Therefore, it is urgently necessary to integrate habitat suitability modeling with evaluations of phytochemical responses, in order to support the conservation and sustainable utilization of medicinal plant resources in dryland regions.

*Cynomorium songaricum* Rupr. is a precious holoparasitic plant endemic to arid regions, primarily distributed in the desert areas of Central and East Asia, and has been used as both food and medicine by local populations for centuries (Zhang et al., 2023). The host plants of *C. songaricum* are mainly *Nitraria* spp (Zhou et al., 2009). The Pharmacopoeia of the People’s Republic of China 2020 highlights its historical usage primarily for the treatment of impotence, premature ejaculation, and spermatorrhea (Xu et al., 2021). Contemporary pharmacological investigations reveal the presence of phytochemical constituents including phenolic acids, polysaccharides, and triterpenoids in *C. songaricum*, which have the effect of anti-oxidant, anti-viral, anti-obesity, anti-diabetes, anti-Alzheimer, and alleviates of memory impairment (Ma et al., 2010; Chen et al., 2020; Cheng et al., 2021; Wang et al., 2022b; Zhang et al., 2022). The commercial demand for *C. songaricum* herbs has rapidly increased over the years due to its high medicinal values (Wang et al., 2022a). However, migration of suitable habitats and the interference of human community have resulted in degradation of the natural habitats of *C. songaricum*. Currently, *C. songaricum* is classified as a second-level protected plant species in the list of “National Key Protected Wild Plants” (Lu et al., 2022). Although earlier studies have shown that *C. songaricum* growing in desert–steppe and saline–alkali habitats exhibit distinct metabolic profiles (Zhang et al., 2022, Zhang et al., 2024d), the quantitative relationship between environmental variation and *C. songaricum* bioactive components has yet to be reported.

This study aims to address these challenges through two interconnected objectives: 1) Climate-resilient habitat planning: Identify the current and future suitable habitats of *C. songaricum* under climate change, quantify centroid migration of these habitats, and pinpoint stable refugia for conservation prioritization. 2) Quality-driven cultivation zoning: Decipher the nonlinear relationships between environmental factors and the accumulation of key bioactive components using machine learning, thereby delineating regions suitable for high-quality cultivation. These findings will provide the scientific basis for the conservation and sustainable utilization of *C. songaricum* and the planning of high-quality and highly suitable *C. songaricum* planting areas.

## 2 Materials and methods

### 2.1 Sample collection and species occurrence records

A total of 252 C. songaricum samples from 28 sites of the 5 representative production areas in China (Inner Mongolia, Ningxia, Gansu, Qinghai, and Xinjiang) were collected in 2020 and 2021 during field tours, and identified by Prof. Guilin Chen. The baseline distribution data of C. songaricum were obtained from the Global Biodiversity Information Facility, GBIF (https://www.gbif.org/), the Chinese Virtual Herbarium, CVH (https://www.cvh.ac.cn), the National Specimen Information Infrastructure, NSII (http://www.nsii.org.cn), iPlant (https://www.iplant.cn/) and previous literatures (Wang et al., 2022b; Zhao et al., 2023; Wang et al., 2021; Miao et al., 2021) (data access deadline November 2024). To reduce the influence of spatial autocorrelation in species data, we applied a spatial filtering method that selects only one record per 1 km×1 km grid. As a result, we obtained 249 effective occurrence records for model calculations (**Supplementary Figure S1**; **Supplementary Table S1**).

### 2.2 Construction of machine learning models

#### 2.2.1 Environmental variables

The multicollinearity of variables within the same data type may affect prediction accuracy. To address this, pairwise Pearson correlation coefficients (r) were calculated across variable pairs. Following established thresholds in ecological niche modeling, variables with |r| < 0.8 were retained to balance information retention and collinearity control. We selected the variable that was most likely to be related to the growth of *C. songaricum* (Zhang et al., 2024b). The 40 variables selected for modeling included 22 bioclimatic variables, 3 terrain data, and 15 soil factors. All these variables (spatial resolution of 30s and raster data of about 1.0 ×1.0 km^2^) were downloaded from the Worldclim, National Earth System Science Data Center (https://www.geodata.cn), and Earth System Grid Federation (**Supplementary Table S2**).

For predicting future distributions, four shared socioeconomic pathways (SSP126, SSP245, SSP370, and SSP585) were downloaded (EC-Earth3). SSP126 (radiation intensity of 2.6 W/m²) reflects a low-emission scenario, SSP245 (radiation intensity of 4.5 W/m²) reflects a medium-emission scenario, SSP370 (radiation intensity of 7.0 W/m²) represents a high-medium emission scenario, and SSP 585 (radiation intensity of 8.5 W/m²) corresponds to a high-emission scenario (Cheng et al., 2024a). Three periods (2050s, 2070s, and 2090s) were chosen to predict the potential distribution. Moreover, based on the condition that soil and terrain factors remain static over the next few decades, only climatic factors were used for future periods in this study, while soil and terrain factors were used for the current period. Finally, 21 environmental variables were retained to run the model.

#### 2.2.2 MaxEnt model processing

The MaxEnt v3.4.1 software integrates environmental variables and distribution data for predicting species distribution and habitat (Cheng et al., 2024a). The MaxEnt v3.4.1 was parameterized as a 75% training set, 25% test set (Shen et al., 2022). It utilized a “bootstrap” method as a maximal iteration model, with a maximum number of repetitions of 10,000, repeated 10 times. The percent contribution of each environmental variable was evaluated using the jackknife method (Zhang et al., 2019). Model accuracy was evaluated using the receiver operating characteristic (ROC) curve and the area under the curve (AUC), with values of 0.9 < AUC < 1.0 indicating excellent predictive performance (Carrell et al., 2023. The ROC curve generated by the MaxEnt model illustrates an AUC value of 0.956 for the *C. songaricum* distribution model based on 21 environmental variables (**Supplementary Figure S2**).

The suitability was divided into four grades by Jenks’ natural breaks, namely, no suitability (0–0.07), low suitability (0.07–0.24), medium suitability (0.24–0.47), and high suitability (0.47–0.95), to obtain the potential distribution area of *C. songaricum* (Cheng et al., 2024a).

### 2.3 Chemical composition analysis

#### 2.3.1 Chemicals and reagents

3,4-Dihydroxybenzaldehyde, catechin hydrate, epicatechin, ursolic acid and sucrose standards reagents were purchased from Yuanye Bio-Technology Co., Ltd. (Shanghai, China). Ethanol of UPLC grade was supplied by Fisher Scientific (Geel, Belgium). Phosphoric acid and acetic acid were supplied by Aladdin Biochemical Technology Co., Ltd. (Shanghai, China).

#### 2.3.2 Sample preparation and UPLC analysis

The plant materials were air-dried in a cool and dry place within 86.71°–113.32° E and 36.34°–43.08° N, a total of 28 sampling sites were selected. Detailed information on the sampling points can be found in **Supplementary Table S3**. Each group contained three biological replicates, with each replicate sample consisting of a mixture of three *C. songaricum* individuals. The mixed samples were ground into fine powder using a mechanical grinder and sieved through a stainless steel sieve (187.5 μm pore size). Each sample (0.1 g) was mixed with 5 mL methanol and ultrasonicated at 25°C for 40 min. The mixture was then centrifuged at 12,000 rpm for 6 minutes, and the supernatant was collected. Before UPLC analysis, the supernatant was filtered through a 0.22 μm hydrophobic syringe filter.

Chromatographic analysis was performed using a Shimadzu UPLC-PDA system (LC-40D xs) equipped with a quaternary solvent pump, autosampler, thermostatted column compartment, and photodiode array detector (PDA). A Shim-pack GIST C18 analytical column (100 mm × 2.1 mm, 2 μm) was used for sample separation. The mobile phase consisted of acetonitrile (A) and 0.3% phosphoric acid solution (B), with a flow rate of 0.2 mL/min. The gradient elution program was as follows: initial conditions of 4.5% A, increasing to 5% A at 5 minutes, 12.5% A at 9 minutes, 17.4% A at 19 minutes, 28% A at 21 minutes, 35% A at 23 minutes, and returning to 4.5% A at 23.5 minutes, followed by equilibration for 5.5 minutes. The column temperature was 25°C, and the injection volume was 10 μL. Analytes, including 3,4-dihydroxybenzaldehyde, catechin, and epicatechin were detected at a wavelength of 230 nm.

For optimal chromatographic separation of ursolic acid, the mobile phase was composed of acetonitrile (A) and 0.1% acetic acid solution (C), with a gradient elution ratio of 85:15 (v/v) for acetonitrile and acetic acid solution. The flow rate was maintained at 0.2 mL/min, the column temperature was 40°C, and the injection volume was 10 μL. Ursolic acid was detected at a wavelength of 210 nm.

#### 2.3.3 Preparation and quantitative analysis of total phenolic samples

Total phenolic were extracted using the method described by Merve et al (Merve et al., 2023). Total phenolics were extracted by adding 20 mL of 63% (v/v) ethanol to 1g of sample powder, followed by ultrasonication at 70°C for 56 minutes. The homogenate was centrifuged at 12,000 rpm for 8 minutes, and the supernatant was stored at –20°C for analysis. Total phenolic were analyzed according to the procedure outlined by Predrag et al (Predrag et al., 2017). For phenolic content determination, 0.1 mL of the extract was mixed with 0.2 mL of Folin–Ciocalteu reagent and 2 mL of distilled water. After standing for 3 minutes at room temperature, 1 mL of 20% (w/v) sodium carbonate was added. The mixture was incubated at 50°C for 25 minutes, and absorbance was measured at 765nm using a Multiskan GO 1510 spectrophotometer (Thermo Fisher Scientific, Finland). Gallic acid (50–250mg/L) was used to generate a standard curve, and total phenolics were expressed as gallic acid equivalents (GAE).

#### 2.3.4 Preparation and quantitative analysis of crude polysaccharide fraction samples

Extraction and analysis of crude polysaccharide fraction were performed with a slight modification to the method described by Wang et al. (Wang et al., 2010). In brief, *C. songaricum* powder (1 g) was boiled in water (1:5 w/v) for 3 hours, repeated three times, followed by precipitation with ethanol at 4°C for 24 hours. The mixture was then centrifuged at 12,000 rpm for 15 minutes. The resulting precipitate was vacuum freeze-dried to obtain crude polysaccharides. Proteins in the crude polysaccharides were removed using the Sevage method (Huang et al., 2010). The crude polysaccharides were washed alternately with ethanol, acetone, and diethyl ether three times to remove lipid residues completely. The phenol-sulfuric acid method (DuBois et al., 1956) was employed to determine the polysaccharide content of the crude polysaccharide fraction.

#### 2.3.5 Method validation

Validation of method for determination of the chemical constituents of *C. songaricum* in terms of linearity, precision, stability and reproducibility (**Supplementary Table S4**).

Linearity: Evaluating the standard solution within a concentration range appropriate for measuring the relevant analyte in the matrix sample allows one to assess the solution’s linearity. The master batch prepared was diluted using methanol to obtain different concentrations of the standard mixture, which was analyzed according to the conditions in 2.3.2. to establish the calibration curve. Each constituent’s mass concentration (x, μg/mL) was measured and linearly regressed on the corresponding peak area (y), to acquire the corresponding regression equations and correlation coefficients. The correlation coefficients for the five major chemical components exceeded 0.990.

Intra-day accuracy: Samples of *C. songaricum* (sample No. MQ1 for ursolic acid, sample No. JLT13 for others) were taken and the peak area RSD_area_% of each component was calculated by injecting the samples six times consecutively in one day, respectively. The intra-day precision ranged from 1.045% to 1.999% (RSD _area_%).

Inter-day accuracy: Samples were injected separately for 3 consecutive days (6 parallel samples on day 1, 3 parallel samples on day 2, and 3 parallel samples on day 3), and the peak area RSD_area_% of each component was calculated. The inter-day precision ranged from 0.939% to 1.941% (RSD _area_%), which was less than 2%, indicating that the instrument’s precision was good.

Stability: The sample solutions of *C. songaricum* were placed at 4°C and then sampled at 0, 2, 4, 6, 8, 10, 12, 18, 24 and 36h. The peak area of each component (RSD_area_%) was calculated. The RSD was less than 2%, indicating that all of the constituents had good stability throughout 36 hours. The stability ranged from 1.225% to 1.895%, with RSD _area_% values all below 2%.

Repeatability: Take 0.1 g powder of *C. songaricum*, parallel 6 groups, and measure respectively. The RSD was less than 3%, representing good reproducibility and stability of the method. The repeatability RSD (%) ranged for peak area (0.526%–2.385%) and retention time (0.066%–0.525%) across the five components.

### 2.4 Statistical analysis

The content of six bioactive components of *C. songaricum* was used for principal component analysis (PCA) on samples collected from 28 sites. Using partial least squares regression (PLSR), we analyzed the correlations between bioactive components of *C. songaricum* and ecological factors, and generated a spatial distribution map of the components concentrations. All PLSR analyses were run on the SPSSPRO cloud platform https://www.spsspro.com/. Additionally, we trained three regression-based machine-learning models in R—Random Forest (randomforest package), Gradient Boosting Decision Tree (GBDT; gbm package), and CatBoost (catboost package)—to identify the environmental factors that most strongly influence the accumulation of bioactive compounds in *C. songaricum*. The statistical analysis was performed using IBM SPSS Statistics version 27 (IBM Corp., Armonk, NY, USA). One-way analysis of variance (One-way ANOVA) was applied to evaluate differences among groups. Mean comparisons were conducted using Duncan’s multiple range test at a significance level of *p* ≤ 0.05.

## 3 Result

### 3.1 Key environmental drivers: BIO13 and pH

Precipitation of wettest month (BIO13) and soil pH 30–60 cm were the two most critical environmental factors influencing the distribution of *C. songaricum*, as indicated by the MaxEnt modeling results. Additional variables, such as sunshine duration in growing season (hsdgs), total phosphorus density in soil (tpd), and max temperature of warmest month (BIO5), also contributed to habitat suitability, but their importance was relatively lower. The relative contributions and permutation importance of these key variables were: BIO13 (27.4%, 12.3%), soil pH (21.9%, 13.2%), hsdgs (8.2%, 12%), tpd (6.9%, 1.5%), and BIO5 (6%, 15.2%) (**Table 1**).

**Table 1:** Detailed information on the 21 ecological factors used for predicting the distribution of *C. songaricum*.

Name	Relative contribution (%)	Permutation importance (%)	Description
BIO13	27.4	12.3	Precipitation of wettest month
pH	21.9	13.2	Soil pH 30–60 cm
hsdgs	8.2	12.0	Sunshine duration in growing season
tpd	6.9	1.5	Total phosphorus density in soil
BIO5	6.0	15.2	Max temperature of warmest month
BIO2	3.9	2.9	Mean diurnal range (mean of monthly (max temp - min temp))
btslt	3.8	2.7	Soil silt content
BIO19	3.6	7.7	Precipitation of coldest quarter
cf	3.3	3.5	Coarse fragment (diameter>2 mm)
BIO15	2.8	2.9	Precipitation seasonality (coefficient of variation)
BIO6	2.5	3.7	Min temperature of coldest month
cec	2.2	3.6	Cation exchange capacity of soil
SOC	1.7	5.4	Soil organic carbon
bd	1.5	1.6	Bulk density of soil
slope	1.3	1.9	Slope gradient
tn	1.0	3.8	Total nitrogen in soil
aspect	0.6	0.6	Aspect
tk	0.6	1.0	Total potassium in soil
tp	0.4	3.2	Total phosphorus in soil
BIO7	0.3	0.9	Temperature annual range (BIO5-BIO6)
BIO3	0.3	0.5	Isothermality (BIO2/BIO7) (×100)

Single-factor response curves indicate that *C. songaricum* reaches peak predicted suitability under the following conditions: 38.02 mm precipitation (precipitation of wettest month, BIO13; suitability range 0.00–202.87 mm), soil pH 9.34 (4.50–9.81), 21590 h sunshine (sunshine duration in the growing season, hsdgs; 5982.60–25014.65 h), 0.21 kg m^-^² total soil phosphorus density (tpd; 0.05–0.46 kg m^-^²), and 33.14°C (maximum temperature of warmest month, BIO5; 0.03–45.43°C) (**Supplementary Figure S3**).

### 3.2 Habitat changes under different climate scenarios

#### 3.2.1 Current suitable habitat is concentrated in north-western China

Based on the MaxEnt predictions, we delineated and visualized the potential range of *C. songaricum* under the integrated regional model (**Supplementary Figure S4**). Under current climatic conditions, suitable habitat is concentrated in north-western China. Areas of high suitability are found chiefly in south-western Inner Mongolia (Ordos, Wuhai, Bayannur and Alxa); north-eastern Gansu (Jiuquan, Jiayuguan, Zhangye, Jinchang, Wuwei, Lanzhou and Baiyin); northern Ningxia (Zhongwei, Wuzhong, Yinchuan and Shizuishan); central Qinghai (Haixi and Hainan); and north-western Xinjiang (Hami, Bayingolin, Hotan, Changji, Tacheng, Bortala, Korgas, Ili, Aksu, Kizilsu and Kashgar).

Nationwide, the total area classified as suitable amounts to 21.90 × 10^5^ km², comprising 2.70 × 10^5^ km² of high-suitability habitat, 6.37 × 10^5^ km² of medium suitability and 12.83 × 10^5^ km² of low suitability—together representing 22.81% of China’s land surface (**Supplementary Table S5**).

#### 3.2.2 Projected suitable habitat areas are expected to increase

**Figure 1:**
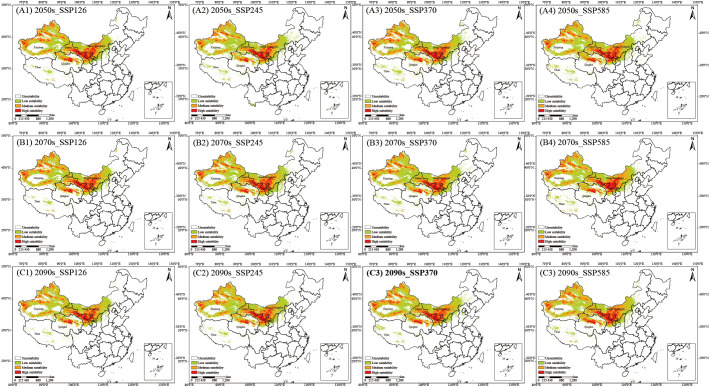
Habitat suitability for *C. songaricum* in China under four emission scenarios: 2050s **(A1–A4)**, 2070s **(B1–B4)**, and 2090s **(C1–C4)**.

*C. songaricum* is primarily distributed in desert regions, among Earth’s most fragile ecosystems. Under the SSP126, SSP245, SSP370, and SSP585 scenarios, the MaxEnt model was applied to predict the potential suitability habitats for *C. songaricum* in the 2050s, 2070s, and 2090s. **Figure 1** depicts the spatial distribution of predicted suitable habitats for *C. songaricum* in the future, categorized into unsuitability, low suitability, medium suitability, and high suitability habitats. High-suitability habitat areas ranged from 33.15 to 39.20 × 10^4^ km² across future periods. (**Figure 1**) (**Supplementary Table S5**). In the 2050s, SSP585 projected the largest high-suitability area (39.63 × 10^4^ km²), while SSP245 projected the smallest (33.15 × 10^4^ km²). For the 2070s, SSP585 again yielded the maximum extent (39.48 × 10^4^ km²), while SSP370 produced the minimum (35.66 × 10^4^ km²). In the 2090s, the greatest area was predicted under SSP585 (40.60 × 10^4^ km²) and the least under SSP126 (34.36 × 10^4^ km²). High-suitability habitats are projected to shift northward under future climate scenarios.

**Figure 2:**
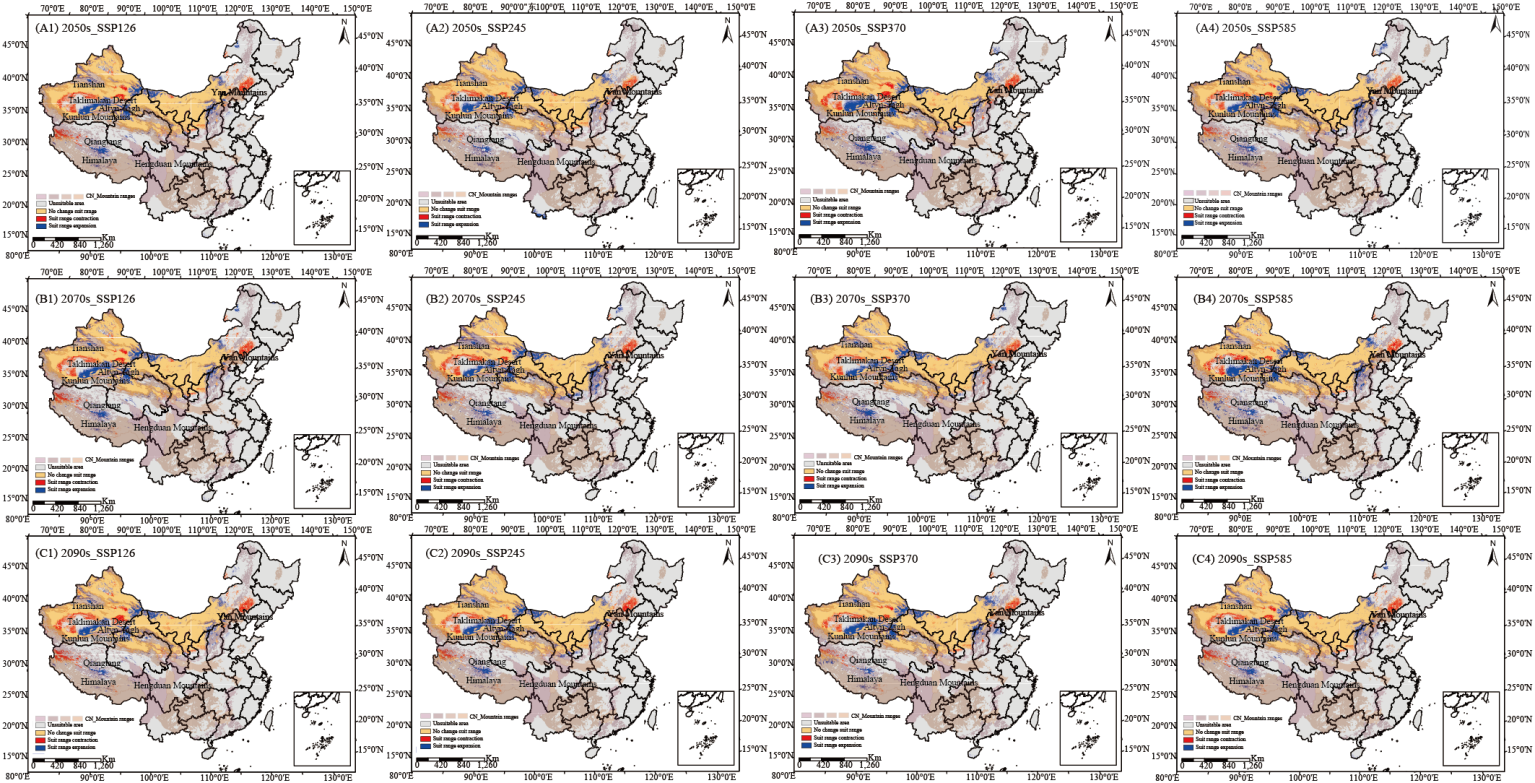
Geographic and spatial pattern changes of overall suitable areas under four emission scenarios in the 2050s **(A1–A4)**, 2070s **(B1–B4)**, and 2090s **(C1–C4)** compared to the current period (blue: expansion areas, yellow: stable areas, red: contraction areas).

Projected suitable habitat areas for *C. songaricum* expand under all future scenarios (**Figures 2A–C**; **Supplementary Table S5**). Suitable habitat gains concentrate along relatively moist mountain and plateau margins, specifically the Altyn-Tagh Fault zone, the southeastern Qiangtang Plateau, the eastern Tianshan Mountains, and the flanks of the Yinshan Mountains. Losses, by contrast, are anchored in hyper-arid deserts and adjacent transition belts, including the western and northern Taklamakan Desert, the Taklamakan–Kumtag ecotone, the north-western Qiangtang Plateau, and the north-eastern Yanshan Mountains. By the 2090s, projected increases in suitable habitat areas are 8.03, 14.42, 19.58, and 23.38 × 10^4^ km² under SSP126, SSP245, SSP370, and SSP585 scenarios, respectively (**Figure 2C**; **Supplementary Table S5**).

Overall, it is evident that future climate warming will positively impact the suitable habitats for *C. songaricum*, as reflected in the lowest habitat expansion under the SSP126 scenario. **Figure 2** illustrates the dynamic changes in the habitat distribution of *C. songaricum*. Tabulated data confirm these distributional shifts under different climate scenarios (**Supplementary Table S5**). In the long term, the four future climate scenarios are projected to increase in highly and moderately suitable habitats for *C. songaricum*, with an average increase of 10.91 × 10^4^ km² and 12.56 × 10^4^ km², respectively.

#### 3.2.3 Centroids migration of suitable areas is minor

**Supplementary Figure S5** shows the centroid position and shift direction for *C. songaricum* under each time slice and SSP scenario, calculated in ArcGIS. The current centroid of the suitable habitat is located in Guazhou County, Jiuquan City, Gansu (95.353°E, 40.622°N). Under future scenarios, centroids shifted only 2.83–32.58 km from this position, remaining within Guazhou County. Overall, the centroid migration of suitable habitat remains minor across all four scenarios.

### 3.3 Ecological quality indicator development

#### 3.3.1 Significant differences in the bioactive component contents among populations

Using UPLC-PDA technology, the contents of four major bioactive components (3,4-dihydroxybenzaldehyde, catechin, epicatechin, and ursolic acid) in 84 samples from 28 C*. songaricum* populations were analyzed. The study also measured the contents of two total components, including total phenolics and crude polysaccharides, revealing variations among them (**Supplementary Table S6**). One-way ANOVA detected significant population-level differences in all measured bioactive components (**Supplementary Table S7**). Concentrations across populations spanned the following ranges: catechin 63.2–6314.4 μg g^-^¹, epicatechin 5.3–524.1 μg g^-^¹, 3,4-dihydroxybenzaldehyde 4.3–16.5 μg g^-^¹, ursolic acid 51.4–509.1 μg g^-^¹, total phenolics 28.3–149.9 mg g^-^¹, and crude polysaccharides 21.8–113.8 mg g^-^¹ (**Supplementary Figures S6A–F**).Significant variation was observed, with maximum 3,4-dihydroxybenzaldehyde in DLT (Inner Mongolia), highest catechin and epicatechin in YQ (Xinjiang), and peak ursolic acid and total phenolics in BDJL (Inner Mongolia), and crude polysaccharides were greatest in NYG (Inner Mongolia).

**Figure 3:**
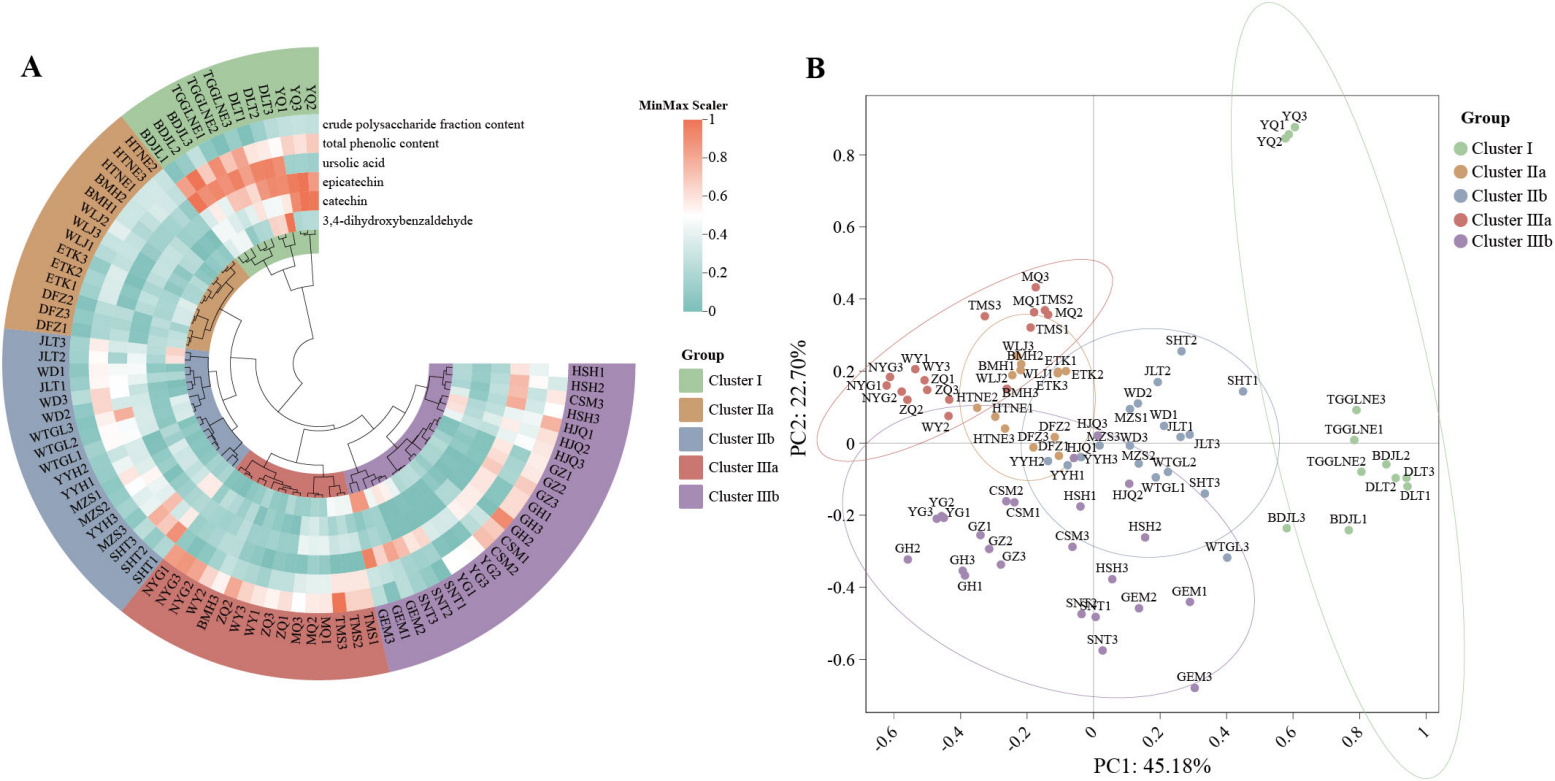
Clustering heatmap and PCA of *C songaricum* **(A)**clustering heatmap of the 9 main components **(B)** PCA plot.

Heatmap clustering (**Figure 3A**) classified samples into five distinct chemical profiles: Cluster I with elevated catechin, epicatechin, ursolic acid, and phenolics; Cluster IIa uniformly low across components; Cluster IIb predominantly elevated ursolic acid and phenolics; Cluster IIIa rich in polysaccharides; and Cluster IIIb with combined elevated ursolic acid and polysaccharides. PCA clearly separated populations along the first two principal components (R² = 0.63, p = 0.001; **Figure 3B**).

#### 3.3.2 Ecological factors affecting bioactive components

**Figure 4:**
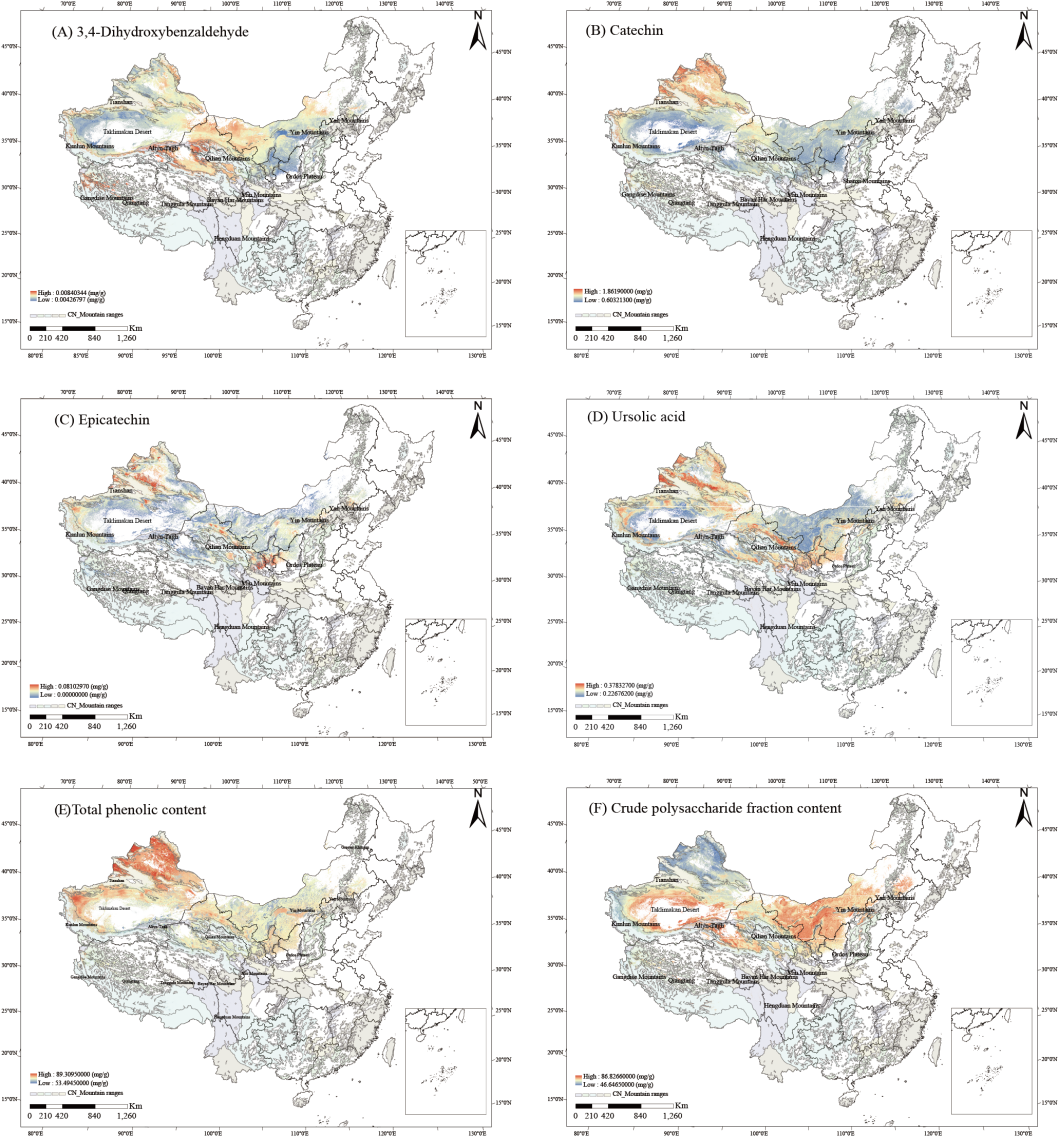
Spatial trends of six bioactive components contents in C songaricum **(A)**. 3,4-dihydroxybenzaldehyde, **(B)** catechin, **(C)** epicatechin, **(D)** ursolic acid, **(E)** total phenolics, **(F)** crude polysaccharides).

The relationships between the contents of six bioactive components and 18 ecological factors were established using PLSR (**Equations S1**–**S6**). The PLSR-derived relationships between components and ecological factors were visualized as spatial trends via ArcGIS (**Figure 4**). High-content zones (HCZs, ≥50% concentration threshold) were identified for all six components: 3,4-Dihydroxybenzaldehyde HCZs clustered at the boundary between the Gangdise Mountains and the northern Qiangtang Plateau, the northern foothills of the Kunlun Mountains, the southern slopes of the Tianshan Mountains, and the periphery of the Qilian Mountains (**Figure 4A**). Catechin HCZs were prominent around the Tianshan and Kunlun Mountains, the junction of the Gangdise Mountains and the western Qiangtang Plateau, and the northern Qilian Mountains (**Figure 4B**). Epicatechin HCZs dominated areas surrounding the Tianshan Mountains, northern Kunlun Mountains, northern Qilian Mountains, northern Ordos Plateau, and Yinshan Mountains (**Figure 4C**). Ursolic acid HCZs concentrated near the Tianshan, Kunlun, and Qilian Mountains, as well as the northern Ordos Plateau (**Figure 4D**). Total phenolics HCZs covered western and northern Xinjiang, western Tibet, central Qinghai, central and northern Gansu, most of Inner Mongolia and Ningxia, and northern Shaanxi (**Figure 4E**). Crude polysaccharide HCZs centered on the Taklimakan Desert periphery and areas north of the Kunlun and Qilian Mountains (**Figure 4F**).

#### 3.3.3 Machine learning for ranking bioactive component drivers

**Figure 5:**
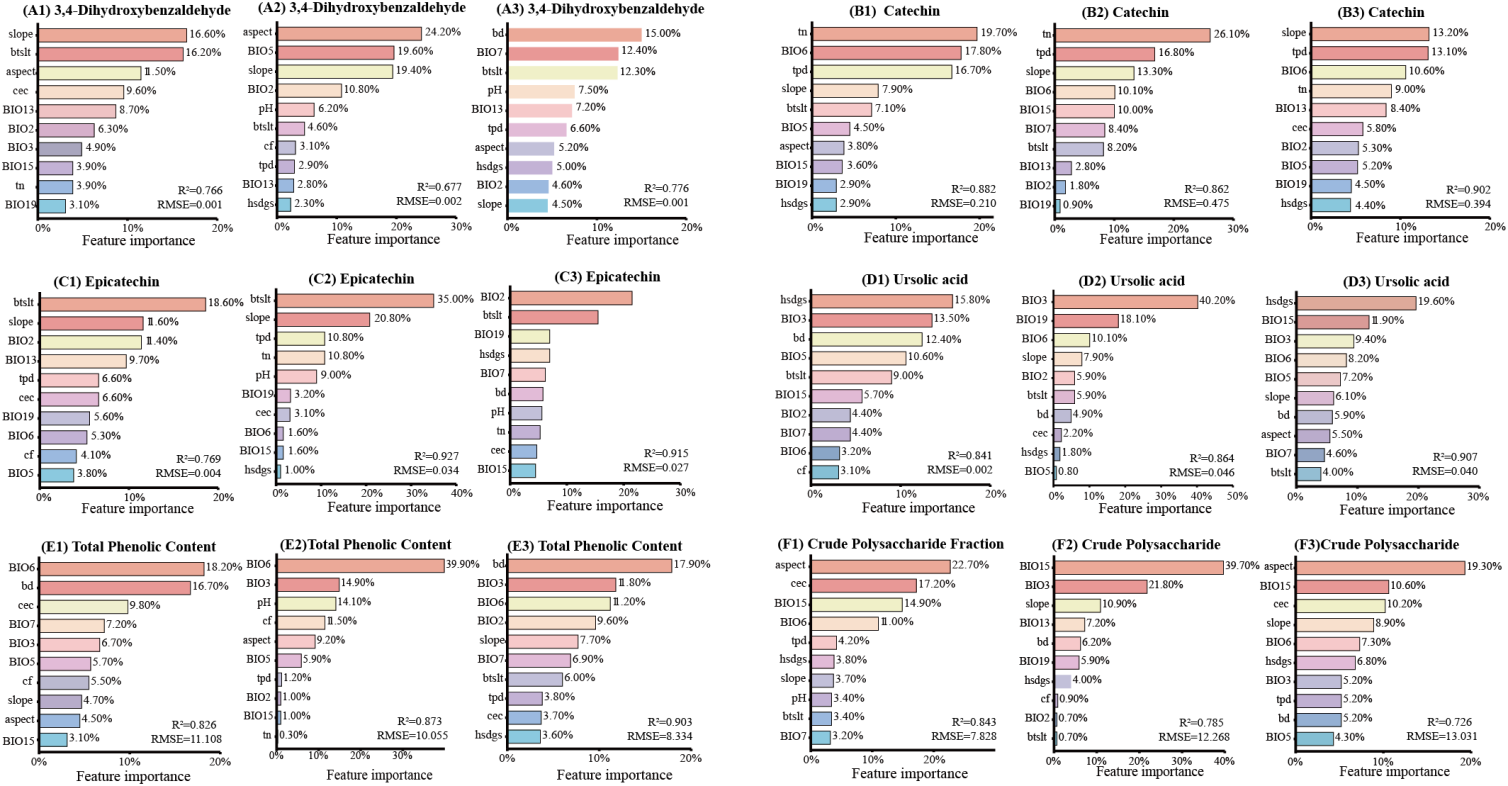
Comparative ranking of climatic factor importance on *C. songaricum* bioactive component accumulation. [From left to right, based on three machine learning methods (random forest, gradient boosting decision tree, and categorical boosting), the top 10 important climatic factors influencing the accumulation of 3,4-dihydroxybenzaldehyde **(A1–A3)**, catechin **(B1–B3)**, epicatechin **(C1–C3)**, ursolic acid **(D1–D3)**, total phenolics **(E1–E3)**, and crude polysaccharides **(F1–F3)**].

To identify the key environmental variables, we applied machine learning models—Random Forest, Gradient Boosting Decision Tree, and Categorical Boosting —to rank variable importance for each bioactive component. For each component, the top 10 influential factors from each model’s ranking were extracted (**Figure 5**), and the intersection of these tri-model top-10 lists was defined as the key environmental factors.

Machine learning identified slope, BIO6, BIO19, hsdgs, and BIO3 as key factors affecting multiple bioactive components (**Figure 5**). For 3,4-dihydroxybenzaldehyde, the core factors were precipitation of wettest month (BIO13), mean diurnal range(BIO2), and slope gradient (slope). Catechin accumulation was primarily influenced by slope, total phosphorus density in soil (tpd), min temperature of coldest month (BIO6), total nitrogen in soil (tn), and precipitation of coldest quarter (BIO19), while the key factors for epicatechin were BIO19 and cation exchange capacity of soil (cec). Ursolic acid accumulation was associated with sunshine duration in growing season (hsdgs), isothermality (BIO3), BIO6, max temperature of warmest month (BIO5), bulk density of soil (bd), and soil silt content (btslt). Total phenolics were strongly influenced by BIO3 and BIO6, whereas crude polysaccharides were influenced by precipitation seasonality (BIO15), slope, and hsdgs.

#### 3.3.4 Identifying high-quality cultivation areas

Based on the study objectives, ArcGIS software was used to identify high-quality regions by superimposing two spatial criteria: high-content zones (HCZs, ≥50% concentration threshold) for individual bioactive components; high-suitability areas (HSAs, 0.47-0.95) predicted by the MaxEnt model. The overlapping zones between HCZs and HSAs were visualized in **Supplementary Figure S7**. The results showed that: the 3,4-dihydroxybenzaldehyde HCZ-HSA overlap covered 14.25×10^4^ km² (**Supplementary Figure S7A**); catechin HCZ-HSA overlap covered 8.76×10^4^ km² (**Supplementary Figure S7B**); epicatechin HCZ-HSA overlap covered 15.05×10^4^ km² (**Supplementary Figure S7C**); ursolic acid HCZ-HSA overlap encompassed 17.60×10^4^ km² (**Supplementary Figure S7D**); total phenolics HCZ-HSA overlap covered 14.03×10^4^ km² (**Supplementary Figure S7E**); crude polysaccharide HCZ-HSA overlap spread across 18.28×10^4^ km² (**Supplementary Figure S7F**).

High-quality cultivation areas included Hotan and Aksu (Xinjiang); Haixi (Qinghai); Jiuquan, Jiayuguan, Zhangye, Jinchang, Wuwei, Baiyin (Gansu); Zhongwei, Wuzhong, Yinchuan, Shizuishan (Ningxia); and Alxa, Bayannur, Ordos (Inner Mongolia) (**Supplementary Figure S7**).

## 4 Discussion

### 4.1 Key environmental factors impacting distribution

MaxEnt modeling accurately predicts species habitats and effectively assesses relationships between species distribution and environmental variables (Li et al., 2020; Liu et al., 2021; Chen et al., 2024; Hosseini et al., 2024). In this study, the MaxEnt model was used to predict the habitat area of *C. songaricum*, achieving an AUC value of 0.956, which indicates high reliability in the prediction results and exceeds the AUC value (0.937) reported in previous studies (Lu et al., 2022). The higher AUC value (0.956 vs. 0.937) suggests that incorporating soil factors, terrain data, and phenology-related environmental variables enhanced the model’s predictive capacity, likely due to a more comprehensive characterization of *C. songaricum*’s ecological requirements compared to previous environmental variable selections. The incorporation of additional critical environmental factors can enhance the accuracy of MaxEnt modeling (Phillips et al., 2006; Bradie and Leung, 2017). Therefore, we incorporated a broader range of critical environmental factors into the model. Previous studies indicate that *C. songaricum* primarily inhabits salt-alkaline soils or desert terrains (Zhang et al., 2022). Significant physicochemical differences (e.g., salinity, pH) across soil types (e.g., sandy soils, saline soils) directly shape its niche differentiation (Zhang et al., 2024c). Therefore, soil factors are integrated as parameters in our ecological niche model. Additionally, terrain data (e.g., slope, elevation) in arid regions modulate microhabitat conditions by redistributing moisture and altering local temperatures, justifying their inclusion as model parameters (McNichol et al., 2024). Notably, phenology-related environmental variables (e.g., seasonal temperature fluctuations), critical for plant growth (He et al., 2023).Above factors overlooked in prior research of *C. songaricum* (Lu et al., 2022), are incorporated here. By integrating these factors, our predictions diverge significantly from earlier models (Lu et al., 2022). In this study, we used the jackknife test in the MaxEnt model to evaluate the bioclimatic variables influencing the geographic distribution of *C. songaricum*.

The results indicate that soil pH is one of the primary environmental drivers influencing the distribution of *C. songaricum*. This study found that the most suitable soil pH value for *C. songaricum* was 9.34, suggesting a preference for alkaline environments. Interestingly, the preference for alkaline and arid conditions may also be related to the ecological traits of its host plant, the genus *Nitraria*, which is commonly found in saline and drought-prone environments (Cheng et al., 2015; Wu et al., 2023). Although high salinity is typically associated with reduced photosynthesis and inhibited growth, previous studies have shown that seedlings of *Nitraria sibirica* can not only tolerate saline–alkali soils but may even exhibit enhanced growth under certain salt concentrations (Wu et al., 2023). This may help explain the single-factor response curves indicating an optimal pH of 9.34 and a relatively low wettest-month precipitation (38.02 mm) for the growth of *C. songaricum*. Another possible explanation is that soil pH not only affects nutrient uptake by plants but also significantly reshapes the structure of rhizosphere microbial communities (Wang et al., 2021). Rhizosphere microorganisms can enhance plant resistance to pathogens and improve plant survival under adverse conditions such as drought and saline–alkali stress (Zhang et al., 2024d). Previous studies have shown that in saline–alkali environments, limited resource availability in the rhizosphere of *C. songaricum* promotes intense bacterial competition, resulting in a higher proportion of negative correlations within the microbial network (Zhang et al., 2024d). Such negative interactions can stabilize microbial communities against external disturbances and enhance network stability under fluctuating conditions, thereby indirectly improving plant survival in extreme environments. In addition, this study found that precipitation during the wettest month (BIO13) also had a significant impact on the distribution of *C. songaricum*. A likely explanation is that precipitation affects rhizosphere microbial dynamics, which in turn influence the growth and survival of the plant. In dryland ecosystems, precipitation typically occurs in pulses, which can trigger short-term surges in soil microbial metabolic activity (Wang et al., 2022c). Rewetting events rapidly reactivate microbial communities and promote the release of inorganic nutrients such as nitrogen and phosphorus. However, if the timing of plant nutrient uptake is not synchronized with microbial nutrient release, nutrients may be lost through leaching or volatilization, reducing overall system efficiency. A moderate level of BIO13 (e.g., the optimal value of 38.02 mm identified in this study) may represent a window of water availability in which microbial activity and plant nutrient uptake are temporally aligned, thereby maximizing nutrient use efficiency and creating favorable conditions for plant growth. In contrast, extreme increases in precipitation may disrupt this balance and potentially constrain the expansion potential of *C. songaricum*. These insights enhance our understanding of how *C. songaricum* adapts to varying habitat conditions.

### 4.2 Spatial pattern shifts under future climate scenarios

Under future climatic conditions, the suitable habitat of *C. songaricum* is projected to expand in the 2050s, 2070s, and 2090s across four climate scenarios (SSP126, SSP245, SSP370, and SSP585). Similar trends have been observed in other medicinal plants, such as *Astragalus mongoliae* or *Astragalus membranaceus* (Wen et al., 2024), *Angelica dahurica* (Zhang et al., 2024a), and *Homonoia riparia* (Yi et al., 2016). Studies have shown that greenhouse gas induced global warming may lead to increased surface aridity and more droughts in the twenty-first century due to decreased precipitation and increased evaporative demand associated with higher vapor pressure deficit under warmer temperatures (Dai et al., 2018). Reduced precipitation may exacerbate drought and reduce soil moisture (Tariq et al., 2024), while increased evapotranspiration may elevate soil pH (Ouyang et al., 2024) ultimately favoring *C. songaricum* growth. This trend is particularly evident in the SSP585 scenario, which exhibits the largest increase in suitability habitats. Unlike prior predictions by Lu et al (Lu et al., 2022), which suggested minimal changes in *C. songaricum*’s potential distribution under future scenarios, our study predicts habitat expansion under all future scenarios and periods. This discrepancy likely stems from our inclusion of more comprehensive distribution and environmental data than earlier models (Phillips et al., 2006; Bradie and Leung, 2017).

The habitat expansion areas are primarily located near the Altyn-Tagh fault zone, southeastern Qiangtang Plateau, eastern Tianshan Mountains, and areas surrounding the Yinshan Mountains. In contrast, habitat contraction areas are mainly found in the western and northern regions of the Taklamakan Desert, the connection zone between the Taklamakan and Kumtag deserts, the northwestern Qiangtang Plateau, and the northeastern Yanshan Mountains. A possible explanation is that while the expanding areas are also classified as arid to semi-arid, they receive more precipitation and exhibit higher humidity compared to the contracting areas. The contracting regions are typically characterized by extreme aridity with minimal effective rainfall. Expanding areas are often located along plateau edges or mountain ranges, featuring complex and diverse topography with varied microclimate conditions. In contrast, the contracting areas are primarily deserts or high-altitude desert lands, characterized by uniform terrain and simpler ecosystems. The expanding regions are likely to support a greater diversity of drought-tolerant plants, forming relatively rich vegetation communities that provide more host options for *C. songaricum*. On the other hand, contracting areas exhibit extremely sparse vegetation and low ecological carrying capacity, making it challenging to sustain the survival of *C. songaricum*.

In all emission scenarios, the area of high-suitability habitats consistently exceeds current levels. This indicates climate warming positively drives habitat suitability by alleviating low-temperature constraints and expanding suitable areas (Cheng et al., 2024b). This effect is particularly pronounced in regions with strong low-temperature limitations, such as high-latitude or high-altitude areas, where habitat suitability significantly improves with climate warming. The fluctuations in suitable areas observed in the 2050s and 2070s suggest non-linear growth influenced by the complex dynamic changes of climatic factors. Uneven spatial and temporal precipitation distribution and high-temperature stress may be the primary contributors (Ding et al., 2024). In high-emission scenarios (SSP585), despite potential increases in extreme climate events such as heatwaves and droughts, the area of suitability habitats continues to grow, reflecting long-term improvements in low-temperature-constrained regions.

The ecological theory of medicinal plants emphasizes the fundamental role of authentic production areas, asserting that the origin determines plants characteristics, medicinal properties, and intrinsic quality (Zhang et al., 2024b). The distribution centroid of *C. songaricum* remains relatively stable, primarily located in Guazhou County, Gansu Province. Gaining deeper insights into the delineation of production areas for *C. songaricum* and examining quality differences across various regions is crucial. This should be considered a paramount direction for exploration, as it will facilitate the selection of high-value cultivation areas.

### 4.3 The relationship between the chemical components and environmental factors

The relationship between environmental shifts and secondary metabolism has long been the focus of research in plant biochemistry, physiology and ecology (Xue et al., 2021). Specifically, medicinal crops are used as optimal model species in this field because their pharmacological and economic value is tightly linked to their concentrations of bioactive compounds (Aghaei and Komatsu, 2013; Guo et al., 2013; Cao et al., 2020). Existing studies have shown that factors such as soil, climate, and topography play crucial roles in the accumulation of secondary metabolites in plants (Zhang et al., 2021; Da Silva et al., 2022; Su et al., 2023). However, how these environmental factors mediate the production of bioactive components of *C. songaricum* remain understudied. We employed machine learning models (Random Forest, Gradient Boosting Decision Trees, and CatBoost) alongside Partial Least Squares Regression (PLSR) to analyze the impact of environmental changes on the bioactive components of *C. songaricum*. We found that slope gradient (slope), min temperature of coldest month (BIO6), precipitation of coldest quarter (BIO19), sunshine duration in growing season (hsdgs), and isothermality (BIO3) were critical for the accumulation of various bioactive components in *C. songaricum*. Slope determines soil moisture content and, together with elevation, jointly shapes the local temperature (Ibrahim et al., 2022). These factors may explain the observed decreases in 3,4-dihydroxybenzaldehyde, catechin, and crude-polysaccharide contents. The phenylpropanoid pathway is modulated by abiotic factors—low temperature among them—which can drive the accumulation of various phenolic compounds (Sharma et al., 2019; Salam et al., 2023). This explains why BIO6 promotes higher levels of catechin and total phenolics in *C. songaricum*. Exposure to both UV-B radiation and drought impairs plant growth and health by boosting the production of reactive oxygen species, which damage lipids, proteins, carbohydrates, and DNA (Shoaib et al., 2024). At the same time, UV-B alone can stimulate the accumulation of terpenoid compounds in many plant species (Zhang et al., 2021). This dual effect may explain why hsdgs elevates ursolic-acid levels while reducing crude-polysaccharide content.

As climate change becomes an increasingly pressing concern, it is vital to identify regions highly suitable for producing high-quality *C. songaricum*. Because the compounds are subject to interactive, non-linear regulation by environmental factors, high-content zones (HCZs, ≥50% concentration threshold) do not entirely coincide with high-suitability areas (HSAs, 0.47-0.95). To date, regions simultaneously offering both high suitability and high compound content for *C. songaricum* have rarely been reported. By integrating our PLSR findings with habitat-suitability classifications, we identified the optimal cultivation zones in northwestern China. These results offer valuable references for planning production areas for high-quality *C. songaricum*. In addition, growers can use our findings to introduce *C. songaricum* selectively into regions best suited to the desired compound profile, thereby obtaining *C. songaricum* enriched in a specific single component or in selected classes of components.

## 5 Conclusion

MaxEnt results indicated that precipitation of wettest month (BIO13) and soil pH were key factors influencing the distribution of *C. songaricum*. Under various future emission scenarios, the suitable habitat area for *C. songaricum* is projected to expand, while the distribution centroid remains largely stable. By PLSR, this study revealed the complex relationships between environmental factors and bioactive components, including 3,4-dihydroxybenzaldehyde, catechin, epicatechin, ursolic acid, total phenolics, and crude polysaccharide. Cross-validation using three machine learning models further identified critical environmental factors affecting composition accumulation. Among them, slope gradient (slope) acted as a key shared negative regulator for 3,4-dihydroxybenzaldehyde, catechin, and crude polysaccharides. Min temperature of coldest month (BIO6) served as a key shared positive regulator for catechin and total phenolics, while functioning as a key negative regulator for ursolic acid. Precipitation of coldest quarter (BIO19) was identified as a key shared negative regulator for catechin and epicatechin. Sunshine duration in growing season (hsdgs) acted as a key positive regulator for ursolic acid while negatively regulating crude polysaccharides. Additionally, BIO3 (isothermality) served as a key shared positive regulator for both ursolic acid and total phenolics. Based on machine learning results, strategies involve precise water management combined with targeted fertilization, and selecting regions characterized by higher minimum temperature of coldest month (BIO6) and lower mean diurnal range (BIO2), thereby enabling the production of high-quality *C. songaricum*. In addition, the study employed geographic information system (GIS) tools to combine the distribution characteristics of bioactive components with habitat suitability analysis. This approach identified highly suitable cultivation areas in northwestern China, including Hotan and Aksu (Xinjiang); Haixi (Qinghai); Jiuquan, Jiayuguan, Zhangye, Jinchang, Wuwei, Baiyin (Gansu); Zhongwei, Wuzhong, Yinchuan, Shizuishan (Ningxia); and Alxa, Bayannur, Ordos (Inner Mongolia). While other high-suitability growth areas (compound content < Avg) can be designated as conservation zones for *C. songaricum*.

In conclusion, this study integrated machine learning models and UPLC technology to provide a scientific foundation for the regional optimization of *C. songaricum* cultivation and the efficient production of bioactive components. The findings not only contribute to the sustainable development of the *C. songaricum* industry but also offer valuable insights into the relationship between ecological factors and the quality of traditional Chinese medicine.

## Data Availability

The original contributions presented in the study are included in the article/**Supplementary Material**. Further inquiries can be directed to the corresponding authors.
