# Supplementary material for: Evaluation of the impact of ecological factors on the habitat suitability and bioactive components accumulation of the medicinal holoparasitic plant Cynomorium songaricum using machine learning models
Source: Front Plant Sci. 2025 Jul 17;16:1586682. doi: 10.3389/fpls.2025.1586682 (PMC12310582; doi:10.3389/fpls.2025.1586682)
Supplement: Supplementary file 1 [file DataSheet1.docx]

Supplementary Material

# Supplementary Figures and Tables

For more information on Supplementary Material and for details on the different file types accepted, please see [here](https://www.frontiersin.org/guidelines/author-guidelines#supplementary-material).

# Supplementary Figures


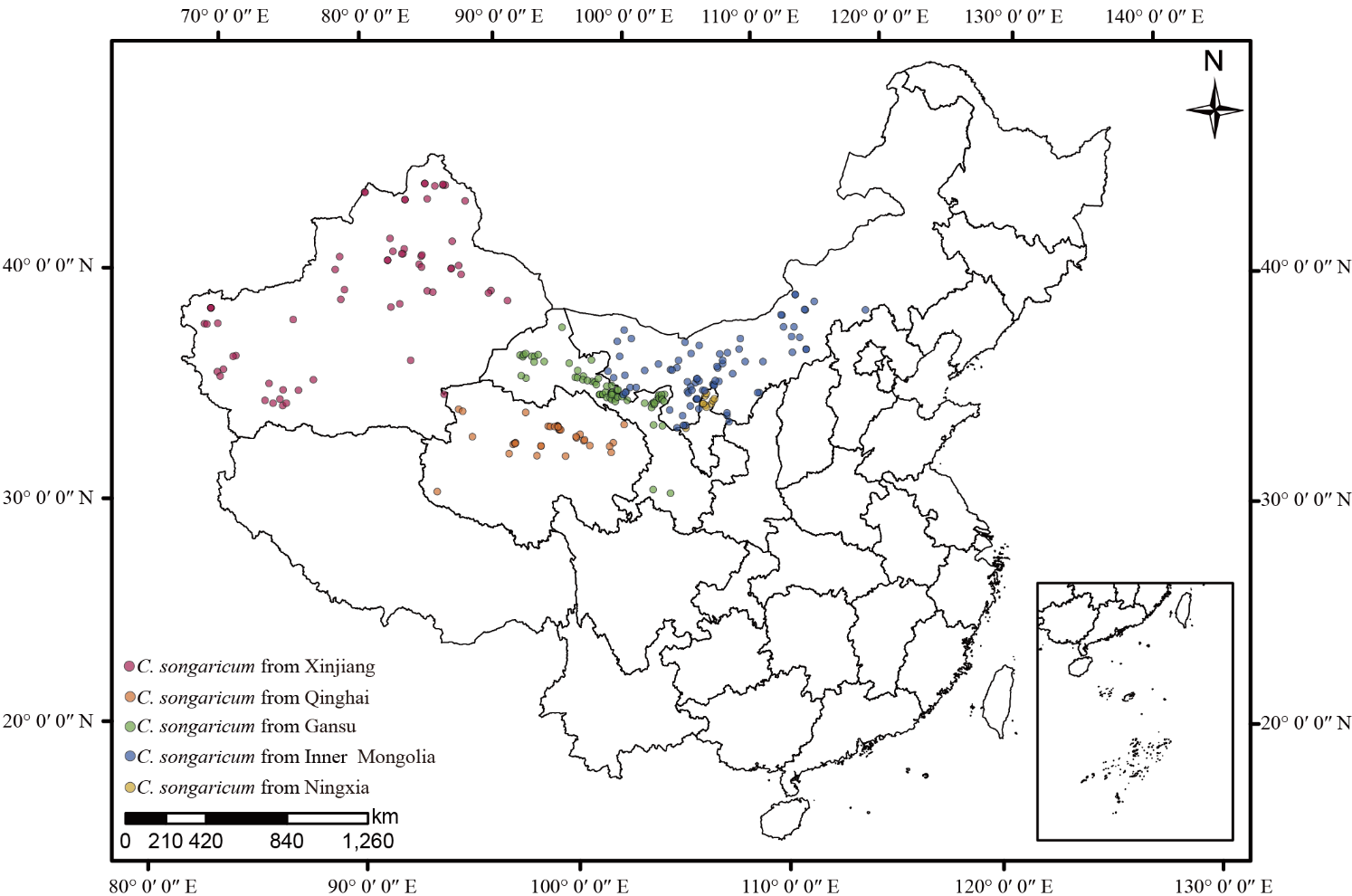


**Figure S1.** Geographic distribution of occurrence data for *C. songaricum.*

**
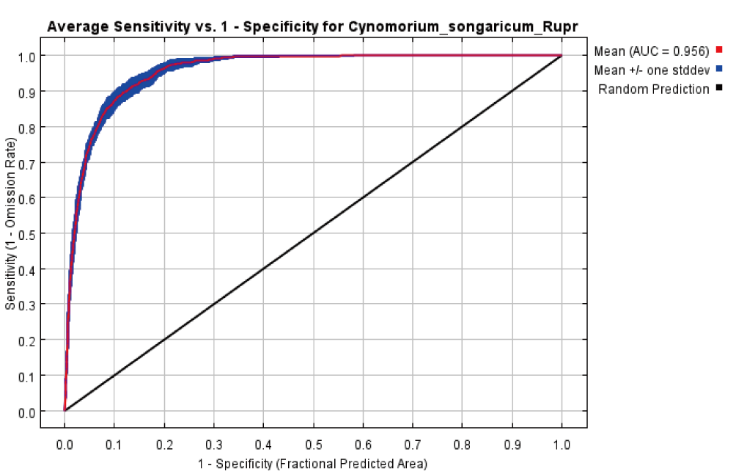
**

**Figure S2.** ROC curve and AUC values from the MaxEnt model predictions. AUC, Area under curve; ROC, Receiver operating characteristic.


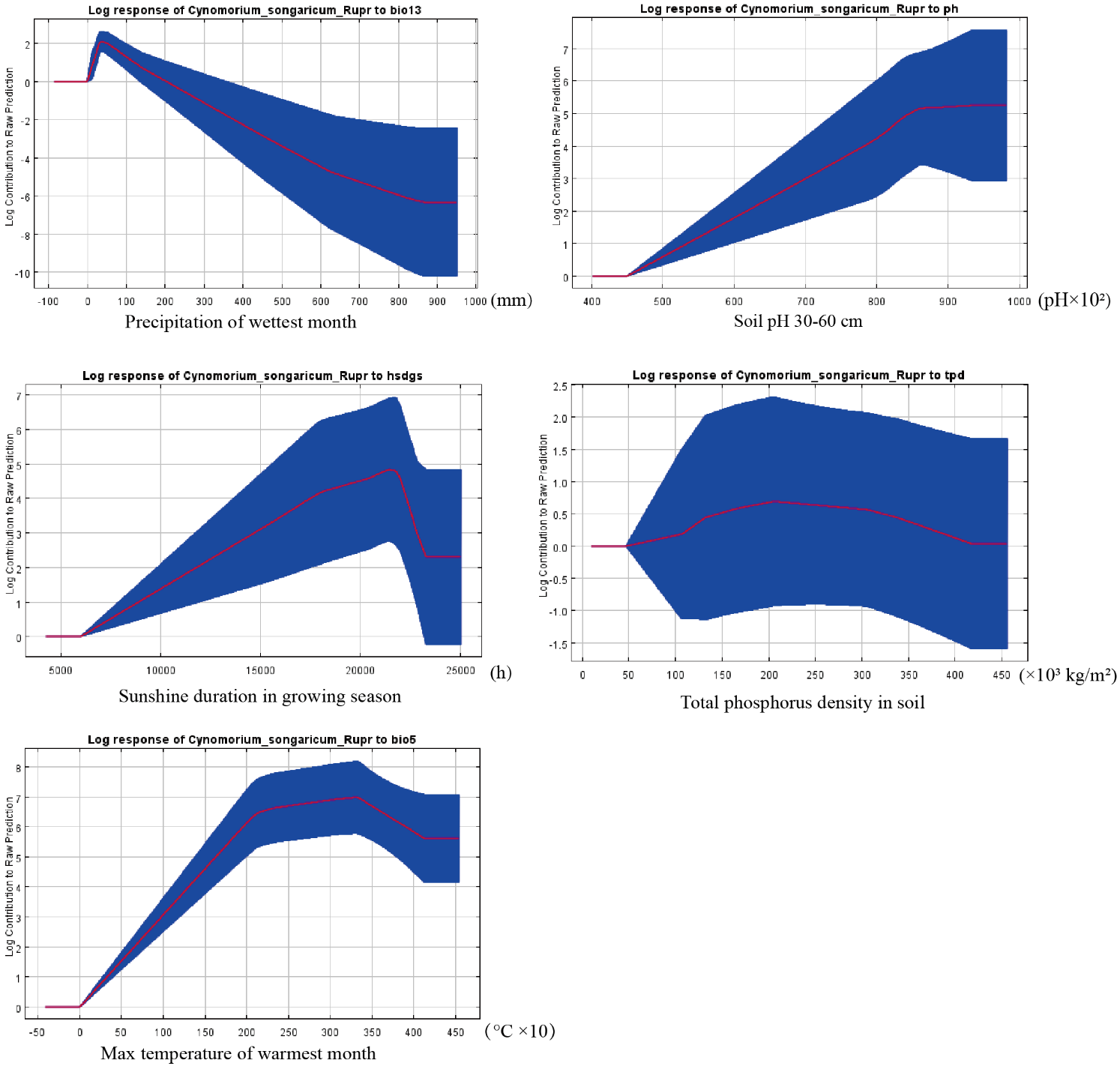


**Figure S3.** Single-factor response curves of major environmental factors affecting *C. songaricum*’s habitat suitability.


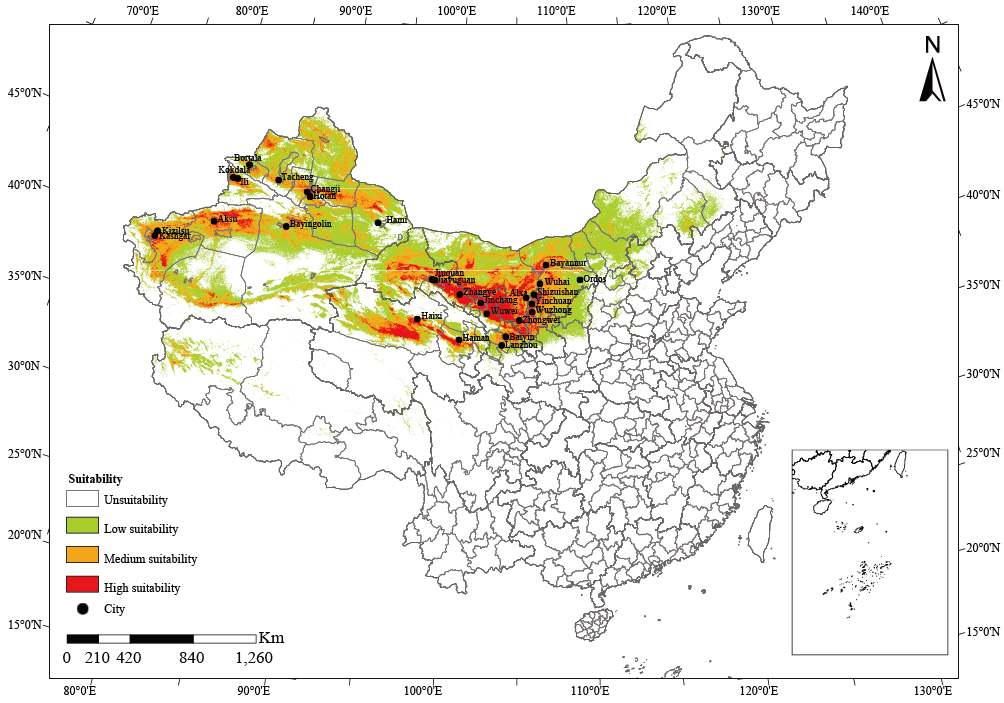


**Figure S4.** Potential suitability habitats for *C. songaricum* in China under current climate scenarios.


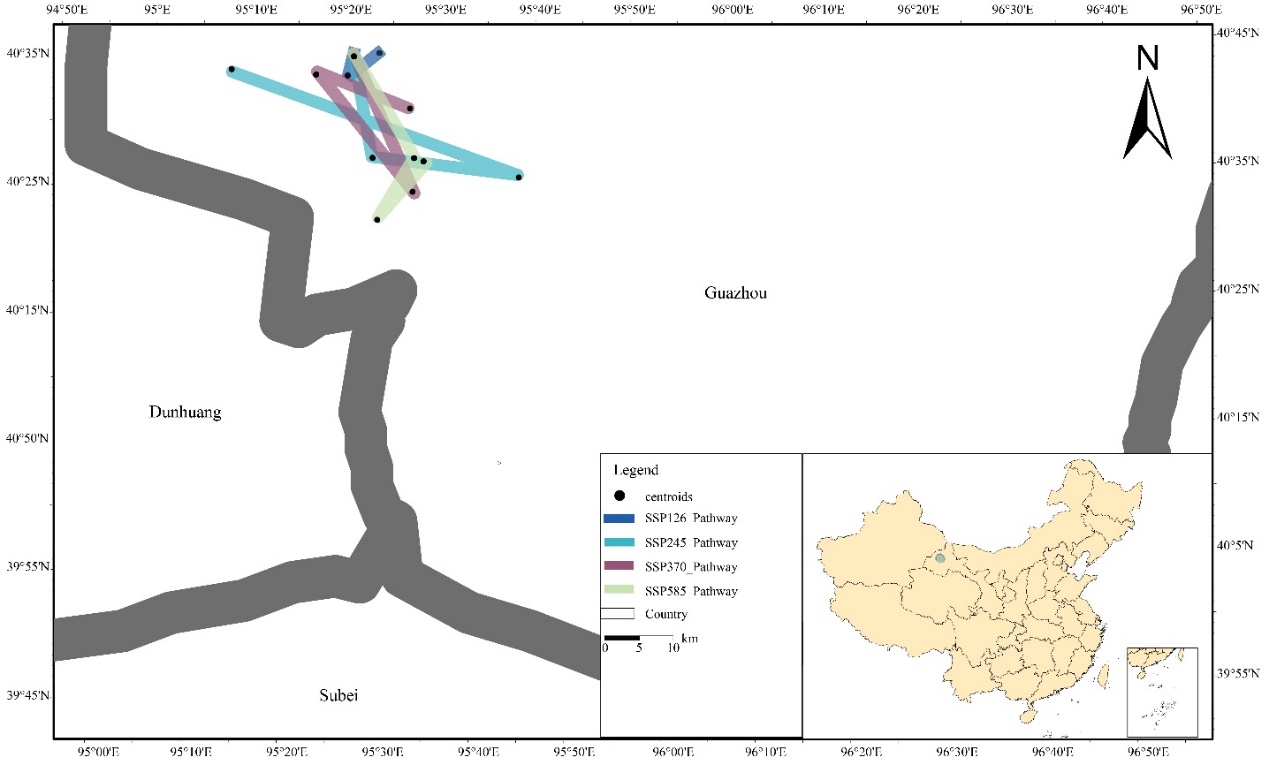


**Figure S5.** Centroids migration of *C. songaricum*’s suitable areas under the future climate scenarios.


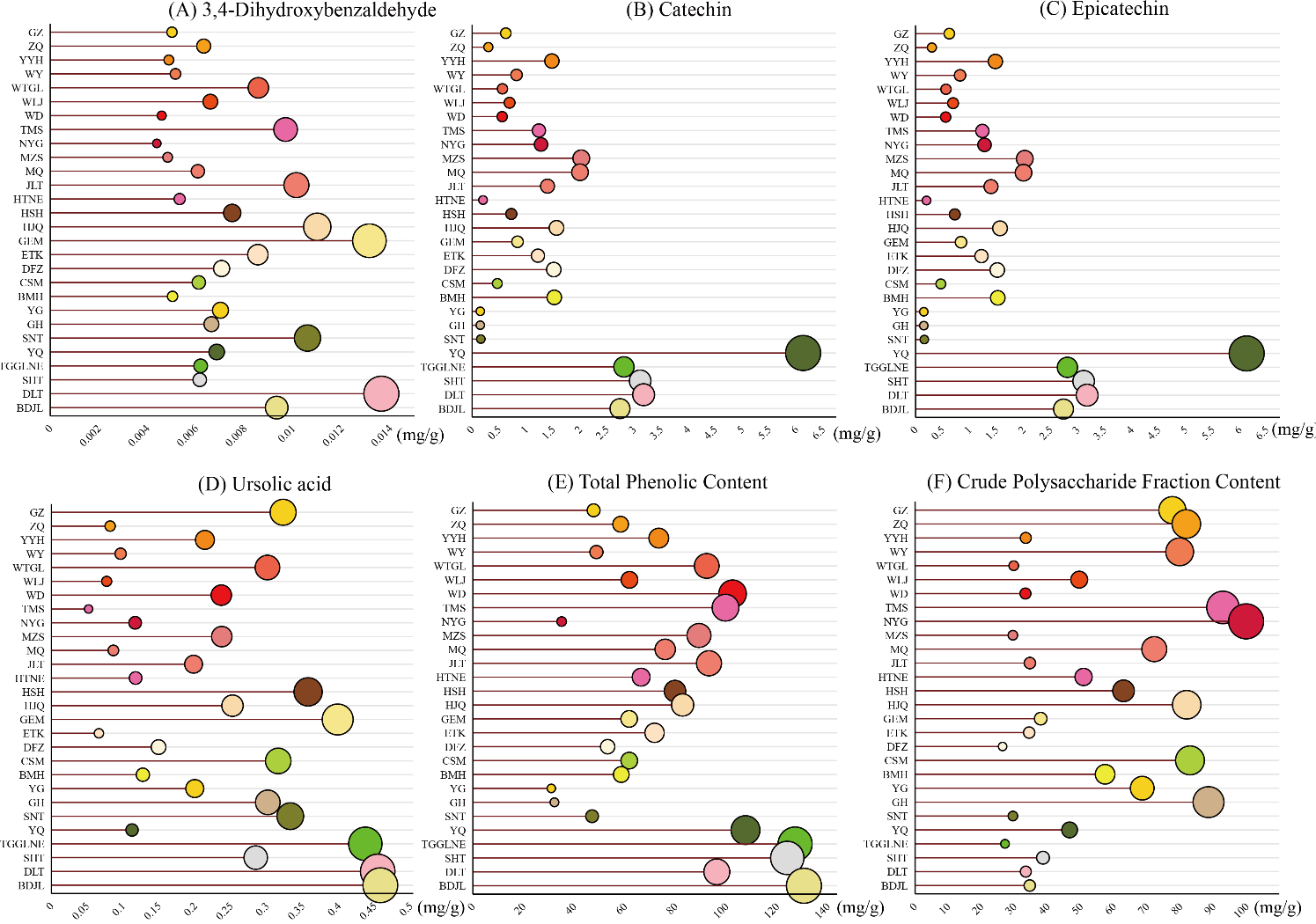


**Figure S6.** Lollipop charts of the contents of six major components in *C. songaricum* (**A.** 3,4-dihydroxybenzaldehyde, **B.** catechin, **C.** epicatechin, **D.** ursolic acid, **E.** total phenolics, **F.** crude polysaccharides).


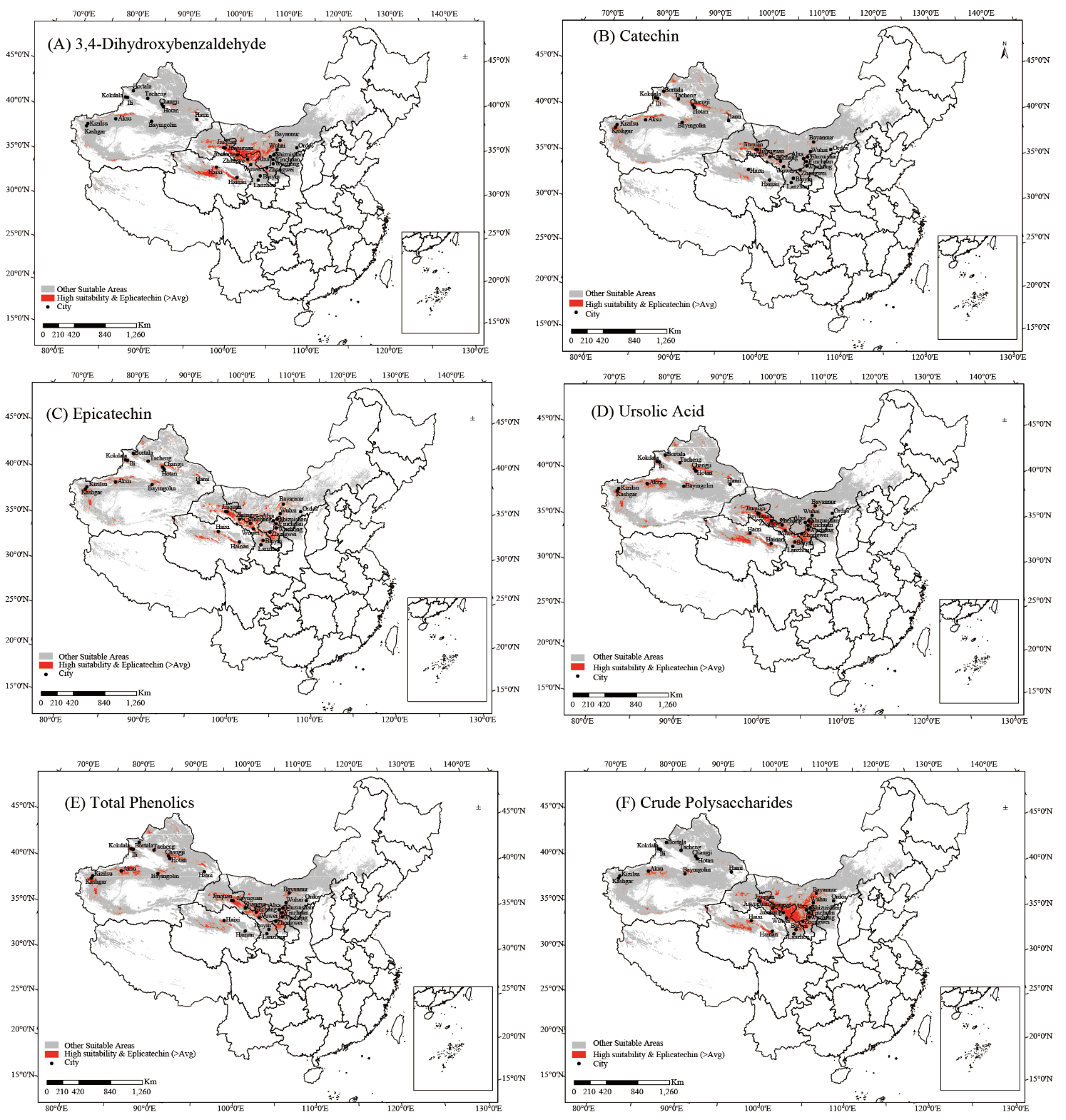


**Figure S7.** Superimposed map of the area of the content of the bioactive components ≥ Avg and the high suitability, with the current climate scenario.

# Supplementary tables

**Table S1.** Longitude and latitude of 249 *C. songaricum* points

| FID | sample | Longitude (E) | Latitude (N) |
| --- | --- | --- | --- |
| 0 | *C. songaricum* | 104.96 | 41.45 |
| 1 | *C. songaricum* | 107.50 | 38.07 |
| 2 | *C. songaricum* | 101.56 | 41.58 |
| 3 | *C. songaricum* | 105.35 | 40.93 |
| 4 | *C. songaricum* | 106.67 | 39.51 |
| 5 | *C. songaricum* | 113.32 | 43.08 |
| 6 | *C. songaricum* | 105.31 | 39.40 |
| 7 | *C. songaricum* | 108.36 | 41.09 |
| 8 | *C. songaricum* | 105.66 | 38.82 |
| 9 | *C. songaricum* | 103.30 | 40.43 |
| 10 | *C. songaricum* | 104.04 | 38.32 |
| 11 | *C. songaricum* | 94.80 | 36.34 |
| 12 | *C. songaricum* | 105.10 | 39.62 |
| 13 | *C. songaricum* | 105.12 | 39.18 |
| 14 | *C. songaricum* | 104.81 | 38.06 |
| 15 | *C. songaricum* | 107.30 | 40.44 |
| 16 | *C. songaricum* | 101.36 | 39.12 |
| 17 | *C. songaricum* | 99.11 | 40.50 |
| 18 | *C. songaricum* | 105.84 | 41.29 |
| 19 | *C. songaricum* | 103.71 | 39.04 |
| 20 | *C. songaricum* | 109.81 | 40.47 |
| 21 | *C. songaricum* | 100.67 | 41.41 |
| 22 | *C. songaricum* | 106.55 | 38.60 |
| 23 | *C. songaricum* | 104.46 | 40.28 |
| 24 | *C. songaricum* | 108.47 | 41.57 |
| 25 | *C. songaricum* | 104.56 | 40.61 |
| 26 | *C. songaricum* | 97.45 | 37.19 |
| 27 | *C. songaricum* | 86.71 | 42.00 |
| 28 | *C. songaricum* | 89.38 | 45.28 |
| 29 | *C. songaricum* | 86.18 | 41.77 |
| 30 | *C. songaricum* | 90.33 | 38.24 |
| 31 | *C. songaricum* | 98.71 | 37.06 |
| 32 | *C. songaricum* | 91.34 | 37.64 |
| 33 | *C. songaricum* | 96.44 | 36.38 |
| 34 | *C. songaricum* | 102.95 | 38.58 |
| 35 | *C. songaricum* | 100.59 | 38.93 |
| 36 | *C. songaricum* | 98.70 | 39.73 |
| 37 | *C. songaricum* | 106.66 | 38.75 |
| 38 | *C. songaricum* | 106.21 | 38.97 |
| 39 | *C. songaricum* | 109.49 | 39.05 |
| 40 | *C. songaricum* | 106.06 | 39.24 |
| 41 | *C. songaricum* | 112.56 | 40.93 |
| 42 | *C. songaricum* | 75.17 | 38.95 |
| 43 | *C. songaricum* | 77.05 | 37.18 |
| 44 | *C. songaricum* | 98.52 | 36.88 |
| 45 | *C. songaricum* | 106.27 | 39.09 |
| 46 | *C. songaricum* | 105.27 | 38.51 |
| 47 | *C. songaricum* | 86.31 | 44.31 |
| 48 | *C. songaricum* | 85.61 | 44.32 |
| 49 | *C. songaricum* | 94.90 | 36.40 |
| 50 | *C. songaricum* | 89.59 | 44.02 |
| 51 | *C. songaricum* | 82.43 | 37.77 |
| 52 | *C. songaricum* | 75.26 | 39.73 |
| 53 | *C. songaricum* | 76.74 | 37.01 |
| 54 | *C. songaricum* | 77.54 | 37.95 |
| 55 | *C. songaricum* | 79.92 | 36.31 |
| 56 | *C. songaricum* | 80.42 | 36.29 |
| 57 | *C. songaricum* | 80.76 | 36.54 |
| 58 | *C. songaricum* | 81.70 | 37.13 |
| 59 | *C. songaricum* | 82.99 | 41.65 |
| 60 | *C. songaricum* | 85.28 | 44.88 |
| 61 | *C. songaricum* | 87.48 | 43.97 |
| 62 | *C. songaricum* | 87.52 | 44.34 |
| 63 | *C. songaricum* | 87.58 | 47.67 |
| 64 | *C. songaricum* | 87.99 | 39.52 |
| 65 | *C. songaricum* | 88.30 | 47.81 |
| 66 | *C. songaricum* | 90.05 | 44.21 |
| 67 | *C. songaricum* | 90.28 | 43.81 |
| 68 | *C. songaricum* | 92.36 | 43.26 |
| 69 | *C. songaricum* | 92.37 | 36.48 |
| 70 | *C. songaricum* | 94.63 | 35.90 |
| 71 | *C. songaricum* | 94.66 | 40.46 |
| 72 | *C. songaricum* | 94.85 | 40.39 |
| 73 | *C. songaricum* | 94.87 | 40.50 |
| 74 | *C. songaricum* | 94.93 | 36.42 |
| 75 | *C. songaricum* | 95.00 | 40.55 |
| 76 | *C. songaricum* | 95.18 | 39.44 |
| 77 | *C. songaricum* | 95.40 | 40.45 |
| 78 | *C. songaricum* | 95.57 | 40.47 |
| 79 | *C. songaricum* | 95.58 | 40.21 |
| 80 | *C. songaricum* | 95.78 | 40.55 |
| 81 | *C. songaricum* | 96.21 | 40.26 |
| 82 | *C. songaricum* | 96.45 | 36.39 |
| 83 | *C. songaricum* | 96.78 | 37.32 |
| 84 | *C. songaricum* | 96.90 | 37.32 |
| 85 | *C. songaricum* | 97.13 | 41.91 |
| 86 | *C. songaricum* | 97.33 | 37.38 |
| 87 | *C. songaricum* | 97.47 | 37.22 |
| 88 | *C. songaricum* | 97.54 | 37.20 |
| 89 | *C. songaricum* | 97.75 | 40.29 |
| 90 | *C. songaricum* | 97.93 | 36.01 |
| 91 | *C. songaricum* | 98.20 | 39.66 |
| 92 | *C. songaricum* | 98.32 | 39.65 |
| 93 | *C. songaricum* | 98.32 | 39.98 |
| 94 | *C. songaricum* | 98.68 | 39.57 |
| 95 | *C. songaricum* | 98.94 | 36.77 |
| 96 | *C. songaricum* | 99.00 | 36.81 |
| 97 | *C. songaricum* | 99.26 | 39.53 |
| 98 | *C. songaricum* | 99.33 | 36.56 |
| 99 | *C. songaricum* | 99.42 | 39.50 |
| 100 | *C. songaricum* | 99.50 | 39.38 |
| 101 | *C. songaricum* | 99.62 | 39.67 |
| 102 | *C. songaricum* | 99.75 | 38.93 |
| 103 | *C. songaricum* | 99.77 | 38.95 |
| 104 | *C. songaricum* | 99.93 | 38.93 |
| 105 | *C. songaricum* | 99.93 | 38.96 |
| 106 | *C. songaricum* | 100.10 | 39.05 |
| 107 | *C. songaricum* | 100.15 | 39.33 |
| 108 | *C. songaricum* | 100.18 | 40.02 |
| 109 | *C. songaricum* | 100.20 | 38.82 |
| 110 | *C. songaricum* | 100.40 | 38.80 |
| 111 | *C. songaricum* | 100.45 | 39.38 |
| 112 | *C. songaricum* | 100.45 | 39.05 |
| 113 | *C. songaricum* | 100.48 | 39.03 |
| 114 | *C. songaricum* | 100.50 | 38.73 |
| 115 | *C. songaricum* | 100.50 | 39.05 |
| 116 | *C. songaricum* | 100.51 | 39.06 |
| 117 | *C. songaricum* | 100.55 | 39.73 |
| 118 | *C. songaricum* | 100.57 | 38.97 |
| 119 | *C. songaricum* | 100.70 | 38.78 |
| 120 | *C. songaricum* | 100.71 | 36.76 |
| 121 | *C. songaricum* | 100.72 | 38.64 |
| 122 | *C. songaricum* | 100.82 | 39.17 |
| 123 | *C. songaricum* | 100.84 | 38.86 |
| 124 | *C. songaricum* | 100.85 | 39.20 |
| 125 | *C. songaricum* | 101.08 | 41.96 |
| 126 | *C. songaricum* | 101.12 | 38.88 |
| 127 | *C. songaricum* | 101.19 | 38.98 |
| 128 | *C. songaricum* | 101.25 | 39.87 |
| 129 | *C. songaricum* | 101.30 | 37.60 |
| 130 | *C. songaricum* | 101.32 | 39.07 |
| 131 | *C. songaricum* | 101.45 | 38.72 |
| 132 | *C. songaricum* | 101.60 | 39.33 |
| 133 | *C. songaricum* | 101.96 | 39.33 |
| 134 | *C. songaricum* | 102.52 | 38.62 |
| 135 | *C. songaricum* | 102.92 | 38.43 |
| 136 | *C. songaricum* | 103.04 | 38.66 |
| 137 | *C. songaricum* | 103.07 | 37.62 |
| 138 | *C. songaricum* | 103.12 | 34.66 |
| 139 | *C. songaricum* | 103.25 | 38.77 |
| 140 | *C. songaricum* | 103.34 | 38.94 |
| 141 | *C. songaricum* | 103.35 | 39.00 |
| 142 | *C. songaricum* | 103.39 | 38.85 |
| 143 | *C. songaricum* | 103.53 | 38.88 |
| 144 | *C. songaricum* | 103.53 | 39.02 |
| 145 | *C. songaricum* | 103.56 | 38.80 |
| 146 | *C. songaricum* | 103.60 | 37.59 |
| 147 | *C. songaricum* | 103.68 | 38.72 |
| 148 | *C. songaricum* | 104.06 | 40.18 |
| 149 | *C. songaricum* | 104.15 | 40.17 |
| 150 | *C. songaricum* | 104.49 | 37.51 |
| 151 | *C. songaricum* | 104.94 | 37.61 |
| 152 | *C. songaricum* | 105.01 | 37.49 |
| 153 | *C. songaricum* | 105.02 | 37.62 |
| 154 | *C. songaricum* | 105.15 | 39.10 |
| 155 | *C. songaricum* | 105.39 | 39.23 |
| 156 | *C. songaricum* | 105.62 | 39.58 |
| 157 | *C. songaricum* | 105.68 | 39.75 |
| 158 | *C. songaricum* | 105.83 | 39.67 |
| 159 | *C. songaricum* | 106.04 | 38.61 |
| 160 | *C. songaricum* | 106.17 | 39.22 |
| 161 | *C. songaricum* | 106.72 | 39.47 |
| 162 | *C. songaricum* | 106.75 | 39.44 |
| 163 | *C. songaricum* | 106.75 | 38.83 |
| 164 | *C. songaricum* | 107.00 | 40.24 |
| 165 | *C. songaricum* | 107.30 | 40.59 |
| 166 | *C. songaricum* | 107.60 | 37.76 |
| 167 | *C. songaricum* | 108.72 | 40.50 |
| 168 | *C. songaricum* | 109.40 | 39.05 |
| 169 | *C. songaricum* | 111.17 | 42.56 |
| 170 | *C. songaricum* | 111.71 | 41.54 |
| 171 | *C. songaricum* | 112.70 | 42.73 |
| 172 | *C. songaricum* | 112.20 | 41.49 |
| 173 | *C. songaricum* | 111.90 | 41.99 |
| 174 | *C. songaricum* | 105.68 | 38.84 |
| 175 | *C. songaricum* | 100.88 | 40.74 |
| 176 | *C. songaricum* | 111.24 | 42.02 |
| 177 | *C. songaricum* | 105.72 | 39.77 |
| 178 | *C. songaricum* | 112.57 | 40.90 |
| 179 | *C. songaricum* | 107.05 | 39.62 |
| 180 | *C. songaricum* | 112.65 | 42.76 |
| 181 | *C. songaricum* | 112.12 | 43.47 |
| 182 | *C. songaricum* | 104.12 | 34.50 |
| 183 | *C. songaricum* | 98.96 | 39.55 |
| 184 | *C. songaricum* | 97.34 | 37.35 |
| 185 | *C. songaricum* | 90.78 | 33.75 |
| 186 | *C. songaricum* | 91.60 | 37.58 |
| 187 | *C. songaricum* | 97.33 | 37.35 |
| 188 | *C. songaricum* | 97.16 | 37.34 |
| 189 | *C. songaricum* | 95.36 | 37.85 |
| 190 | *C. songaricum* | 76.98 | 36.84 |
| 191 | *C. songaricum* | 75.26 | 39.72 |
| 192 | *C. songaricum* | 89.58 | 44.00 |
| 193 | *C. songaricum* | 87.24 | 47.03 |
| 194 | *C. songaricum* | 87.54 | 44.41 |
| 195 | *C. songaricum* | 85.39 | 43.87 |
| 196 | *C. songaricum* | 86.32 | 44.53 |
| 197 | *C. songaricum* | 92.22 | 43.14 |
| 198 | *C. songaricum* | 88.13 | 47.79 |
| 199 | *C. songaricum* | 106.82 | 39.67 |
| 200 | *C. songaricum* | 107.49 | 38.18 |
| 201 | *C. songaricum* | 107.14 | 40.89 |
| 202 | *C. songaricum* | 116.53 | 42.45 |
| 203 | *C. songaricum* | 106.22 | 38.54 |
| 204 | *C. songaricum* | 105.70 | 38.84 |
| 205 | *C. songaricum* | 102.45 | 40.13 |
| 206 | *C. songaricum* | 103.09 | 38.62 |
| 207 | *C. songaricum* | 100.48 | 38.96 |
| 208 | *C. songaricum* | 94.88 | 39.51 |
| 209 | *C. songaricum* | 100.62 | 36.30 |
| 210 | *C. songaricum* | 94.91 | 36.38 |
| 211 | *C. songaricum* | 97.38 | 37.32 |
| 212 | *C. songaricum* | 98.48 | 36.94 |
| 213 | *C. songaricum* | 106.27 | 38.45 |
| 214 | *C. songaricum* | 106.07 | 38.62 |
| 215 | *C. songaricum* | 88.29 | 42.80 |
| 216 | *C. songaricum* | 93.49 | 42.90 |
| 217 | *C. songaricum* | 86.22 | 44.29 |
| 218 | *C. songaricum* | 87.67 | 43.84 |
| 219 | *C. songaricum* | 83.06 | 42.12 |
| 220 | *C. songaricum* | 75.95 | 39.15 |
| 221 | *C. songaricum* | 75.26 | 39.72 |
| 222 | *C. songaricum* | 77.41 | 37.89 |
| 223 | *C. songaricum* | 80.80 | 37.00 |
| 224 | *C. songaricum* | 82.28 | 43.57 |
| 225 | *C. songaricum* | 82.96 | 46.75 |
| 226 | *C. songaricum* | 85.39 | 43.87 |
| 227 | *C. songaricum* | 85.74 | 46.80 |
| 228 | *C. songaricum* | 89.82 | 47.21 |
| 229 | *C. songaricum* | 86.86 | 47.70 |
| 230 | *C. songaricum* | 104.86 | 37.62 |
| 231 | *C. songaricum* | 111.12 | 42.58 |
| 232 | *C. songaricum* | 107.63 | 40.94 |
| 233 | *C. songaricum* | 111.69 | 40.82 |
| 234 | *C. songaricum* | 106.95 | 40.32 |
| 235 | *C. songaricum* | 112.13 | 43.47 |
| 236 | *C. songaricum* | 96.27 | 35.92 |
| 237 | *C. songaricum* | 81.01 | 36.29 |
| 238 | *C. songaricum* | 79.91 | 37.11 |
| 239 | *C. songaricum* | 80.37 | 40.24 |
| 240 | *C. songaricum* | 82.99 | 46.73 |
| 241 | *C. songaricum* | 75.32 | 38.96 |
| 242 | *C. songaricum* | 81.19 | 36.42 |
| 243 | *C. songaricum* | 88.65 | 42.79 |
| 244 | *C. songaricum* | 86.86 | 47.70 |
| 245 | *C. songaricum* | 88.13 | 47.83 |
| 246 | *C. songaricum* | 82.21 | 42.94 |
| 247 | *C. songaricum* | 85.74 | 46.80 |
| 248 | *C. songaricum* | 107.90 | 39.96 |

**Table S2.** List of data information sources.

| Abbreviations | Full description | Data sources | Data used in the article |
| --- | --- | --- | --- |
| GBIF | Global Biodiversity Information Facility | https://www.gbif.org | data on species distribution |
| CVH | the Chinese Virtual Herbarium | https://www.cvh.ac.cn | data on species distribution |
| NSII | National Specimen Information Infrastructure | http://www.nsii.org.cn | data on species distribution |
| iPlant | Plant Plus of China | https://www.iplant.cn | data on species distribution |
| WorldClim | WorldClim Global Climate Data | https://worldclim.org | bioclimatic factors (BIO1 –BIO19) |
| NESSDC | National Earth System Science Data Center | https://www.geodata.cn | terrain data, and soil factors |
|  | Chinese Medicine resources geospatial grid information Database | http://www.tcmresources.com | phenology-related environmental variables |

**Table S3.** The numbers and codes of 252 *C. songaricum* samples from 28 sites of the 5 representative production areas in China included in the study.

| S.No. | Province | Region | Population | Longitude(E) | Latitude (N) | Elevation (m) |
| --- | --- | --- | --- | --- | --- | --- |
| 1 | Inner Mongolia | Alxa League | YG | 104.96 | 41.45 | 800 |
| 2 | Inner Mongolia | Erdos | ETK | 107.50 | 38.07 | 1330 |
| 3 | Inner Mongolia | Alxa League | WTGL | 101.56 | 41.58 | 921 |
| 4 | Inner Mongolia | Alxa League | NYG | 105.35 | 40.93 | 1379 |
| 5 | Inner Mongolia | Wuhai | WD | 106.67 | 39.51 | 1147 |
| 6 | Inner Mongolia | Xilingol League | SNT | 113.32 | 43.08 | 1037 |
| 7 | Inner Mongolia | Alxa League | JLT | 105.31 | 39.40 | 1147 |
| 8 | Inner Mongolia | BayanNur | WY | 108.36 | 41.09 | 1024 |
| 9 | Inner Mongolia | Alxa League | TGGLNE | 105.66 | 38.82 | 1516 |
| 10 | Inner Mongolia | Alxa League | TMS | 103.30 | 40.43 | 1252 |
| 11 | Inner Mongolia | Alxa League | HSH | 104.04 | 38.32 | 1392 |
| 12 | Qinghai | Golmud | GEM | 94.80 | 36.34 | 2902 |
| 13 | Inner Mongolia | Alxa League | SHT | 105.10 | 39.62 | 1252 |
| 14 | Inner Mongolia | Alxa League | HTNE | 105.12 | 39.18 | 1230 |
| 15 | Inner Mongolia | Alxa League | CSM | 104.81 | 38.06 | 1353 |
| 16 | Inner Mongolia | Erdos | HJQ | 107.30 | 40.44 | 1048 |
| 17 | Inner Mongolia | Alxa League | BDJL | 101.36 | 39.12 | 1520 |
| 18 | Gansu | Jiuquan | MZS | 99.11 | 40.50 | 1500 |
| 19 | Inner Mongolia | BayanNur | BMH | 105.84 | 41.29 | 1931 |
| 20 | Gansu | Wuwei | MQ | 103.71 | 39.04 | 1313 |
| 21 | Inner Mongolia | Erdos | DLT | 109.81 | 40.47 | 1007 |
| 22 | Inner Mongolia | Alxa League | DFZ | 100.67 | 41.41 | 1011 |
| 23 | Ningxia | Yinchuan | YYH | 106.55 | 38.60 | 1126 |
| 24 | Inner Mongolia | Alxa League | WLJ | 104.46 | 40.28 | 1419 |
| 25 | Inner Mongolia | BayanNur | ZQ | 108.47 | 41.57 | 1335 |
| 26 | Gansu | Jiuquan | GZ | 95.51 | 40.51 | 1129 |
| 27 | Qinghai | Delingha | GH | 97.45 | 37.19 | 2862 |
| 28 | Xinjiang | Bayingolin Mongolian Autonomous Prefecture | YQ | 86.71 | 42.00 | 1053 |

**Table S4.** Regression equation, precision, repeatability, and stability of four investigated compounds.

| Peak | Components | Linear equations | R^2^ | Precision RSD_area_(%) | | Stability | Repeatability（n=6） |
| --- | --- | --- | --- | --- | --- | --- | --- |
|  |  |  |  | Intra-day (n=6) | Inter-day (n=3) | RSD_area_（%） | RSD_area_（%） |
| 1 | 3,4-dihydroxybenzaldehyde | y = 5E-09x + 7E-05 5 | 0.9996 | 1.5273 | 1.9414 | 1.8949 | 0.5259 |
| 2 | Catechin | y = 1E-08x + 6E-05 | 0.9999 | 1.5878 | 0.9387 | 1.3618 | 2.2273 |
| 3 | Epicatechin | y = 1E-08x - 0.0002 | 0.9962 | 1.0446 | 1.1601 | 1.2255 | 2.3852 |
| 4 | Ursolic acid | y = 5E-08x + 0.0009 | 0.9999 | 1.9986 | 1.5959 | 1.7431 | 0.7376 |

**Table S5.** Temporal changes in suitable habitats of *C. songaricum* under different Shared Socioeconomic Pathways (SSP) scenarios.

| Scenario | period | Hihg Suitability area (×10^4^ km^2^) | Change (%) | Medium Suitability area (×10^4^ km^2^) | Change (%) | Low Suitability area (×10^4^ km^2^) | Change (%) | Total Suitability area (×10^4^ km^2^) | Change (×10^4^ km) |
| --- | --- | --- | --- | --- | --- | --- | --- | --- | --- |
|  | Current | 26.99 |  | 63.72 |  | 128.32 |  | 219.03 |  |
| SSP126 | 2050s | 39.20 | 12.21 | 77.68 | 13.96 | 117.47 | -10.85 | 234.35 | 15.32 |
|  | 2070s | 39.20 | 12.21 | 77.68 | 13.96 | 117.47 | -10.85 | 234.35 | 15.32 |
|  | 2090s | 34.36 | 7.37 | 74.74 | 11.02 | 117.96 | -10.36 | 227.06 | 8.03 |
| SSP245 | 2050s | 33.15 | 6.16 | 72.01 | 8.29 | 130.02 | 1.70 | 235.18 | 16.15 |
|  | 2070s | 39.46 | 12.47 | 76.62 | 12.90 | 132.01 | 3.69 | 248.09 | 29.06 |
|  | 2090s | 35.28 | 8.29 | 76.42 | 12.70 | 121.75 | -6.57 | 233.45 | 14.42 |
| SSP370 | 2050s | 38.53 | 11.54 | 74.03 | 10.31 | 121.45 | -6.87 | 234.01 | 14.98 |
|  | 2070s | 35.66 | 8.67 | 68.94 | 5.22 | 132.01 | 3.69 | 236.61 | 17.58 |
|  | 2090s | 40.25 | 13.26 | 76.61 | 12.89 | 121.75 | -6.57 | 238.61 | 19.58 |
| SSP585 | 2050s | 39.63 | 12.64 | 78.54 | 14.82 | 127.70 | -0.62 | 245.87 | 26.84 |
|  | 2070s | 39.48 | 12.49 | 78.84 | 15.12 | 124.55 | -3.77 | 242.87 | 23.84 |
|  | 2090s | 40.60 | 13.61 | 83.20 | 19.48 | 118.61 | -9.71 | 242.41 | 23.38 |

**Table S6.** Six bioactive components from twenty-eight sites (mg/g).

| populations | samples | 3,4-Dihydroxybenzaldehyde | Catechin | Epicatechin | Ursolic acid | Total phenolic content | crude polysaccharide fraction content |
| --- | --- | --- | --- | --- | --- | --- | --- |
| GZ | GZ1 | 0.0044 | 0.7368 | 0.0189 | 0.3012 | 43.2795 | 73.5300 |
|  | GZ2 | 0.0046 | 0.4945 | 0.0190 | 0.3189 | 48.8906 | 74.6100 |
|  | GZ3 | 0.0060 | 0.6387 | 0.0292 | 0.3470 | 50.5983 | 84.4800 |
|  | Mean | 0.005 | 0.6233 | 0.0224 | 0.3223 | 47.58948 | 77.54 |
| ZQ | ZQ3 | 0.0068 | 0.3978 | 0.0684 | 0.0799 | 59.6248 | 71.5400 |
|  | ZQ2 | 0.0065 | 0.2709 | 0.0774 | 0.0917 | 54.9896 | 96.0800 |
|  | ZQ1 | 0.0057 | 0.2325 | 0.0671 | 0.0733 | 60.1128 | 77.6400 |
|  | Mean | 0.0063 | 0.3004 | 0.0710 | 0.0816 | 58.2424 | 81.7533 |
| YYH | YYH3 | 0.0048 | 2.0701 | 0.0132 | 0.2352 | 75.2383 | 36.4400 |
|  | YYH2 | 0.0045 | 1.4065 | 0.0122 | 0.2028 | 64.3821 | 32.1100 |
|  | YYH1 | 0.0053 | 0.9516 | 0.0175 | 0.2026 | 80.2395 | 31.5400 |
|  | Mean | 0.0049 | 1.4761 | 0.0143 | 0.2135 | 73.2866 | 33.3633 |
| WY | WY3 | 0.0053 | 0.8816 | 0.0727 | 0.0837 | 51.8181 | 81.8800 |
|  | WY2 | 0.0051 | 0.9514 | 0.0904 | 0.1502 | 47.5488 | 81.5300 |
|  | WY1 | 0.0051 | 0.6330 | 0.0866 | 0.0548 | 46.6950 | 75.8200 |
|  | Mean | 0.0052 | 0.8220 | 0.0832 | 0.0962 | 48.6873 | 79.7433 |
| WTGL | WTGL3 | 0.0089 | 0.5763 | 0.1860 | 0.3896 | 94.9990 | 26.3900 |
|  | WTGL2 | 0.0083 | 0.5879 | 0.1867 | 0.2529 | 90.7297 | 27.2200 |
|  | WTGL1 | 0.0085 | 0.5195 | 0.1811 | 0.2593 | 90.8517 | 35.5700 |
|  | Mean | 0.0086 | 0.5612 | 0.1846 | 0.3006 | 92.1935 | 29.7267 |
| WLJ | WLJ3 | 0.0064 | 0.9413 | 0.1540 | 0.0649 | 63.1623 | 48.2100 |
|  | WLJ2 | 0.0069 | 0.5708 | 0.1482 | 0.0990 | 61.3326 | 57.5100 |
|  | WLJ1 | 0.0065 | 0.5661 | 0.1382 | 0.0667 | 60.7227 | 42.7100 |
|  | Mean | 0.0066 | 0.6927 | 0.1468 | 0.0768 | 61.7392 | 49.4767 |
| WD | WD3 | 0.0043 | 0.4727 | 0.1546 | 0.2632 | 107.0751 | 42.8400 |
|  | WD2 | 0.0047 | 0.5668 | 0.1770 | 0.1956 | 106.8311 | 29.0000 |
|  | WD1 | 0.0048 | 0.6284 | 0.2477 | 0.2502 | 93.2913 | 27.9500 |
|  | Mean | 0.0046 | 0.5559 | 0.1931 | 0.2364 | 102.3992 | 33.2633 |
| TMS | TMS3 | 0.0099 | 1.1434 | 0.1587 | 0.0516 | 100.0002 | 113.7800 |
|  | TMS2 | 0.0096 | 1.5949 | 0.1749 | 0.0514 | 99.3903 | 81.8800 |
|  | TMS1 | 0.0096 | 0.9674 | 0.1688 | 0.0520 | 99.3903 | 82.7000 |
|  | Mean | 0.0097 | 1.2352 | 0.1675 | 0.0516 | 99.5936 | 92.7867 |
| NYG | NYG3 | 0.0044 | 1.6768 | 0.0897 | 0.1156 | 30.2277 | 102.2600 |
|  | NYG2 | 0.0042 | 1.3901 | 0.0804 | 0.1296 | 36.2047 | 96.9400 |
|  | NYG1 | 0.0045 | 0.7571 | 0.0999 | 0.1043 | 38.5223 | 100.1000 |
|  | Mean | 0.0044 | 1.2746 | 0.0900 | 0.1165 | 34.9849 | 99.7667 |
| MZS | MZS3 | 0.0050 | 1.7064 | 0.0557 | 0.2191 | 79.3856 | 28.8200 |
|  | MZS2 | 0.0049 | 2.0218 | 0.0698 | 0.2801 | 88.1682 | 30.7700 |
|  | MZS1 | 0.0045 | 2.3309 | 0.0514 | 0.2118 | 99.8782 | 28.8200 |
|  | Mean | 0.0048 | 2.0197 | 0.0590 | 0.2370 | 89.1440 | 29.4700 |
| MQ | MQ3 | 0.0057 | 2.0210 | 0.2272 | 0.0617 | 74.1405 | 67.5500 |
|  | MQ2 | 0.0064 | 2.2477 | 0.2103 | 0.1063 | 76.3361 | 74.1300 |
|  | MQ1 | 0.0061 | 1.7212 | 0.2212 | 0.0905 | 76.9460 | 74.5700 |
|  | Mean | 0.0061 | 1.9967 | 0.2195 | 0.0862 | 75.8075 | 72.0833 |
| JLT | JLT3 | 0.0123 | 1.5439 | 0.1947 | 0.2009 | 96.4628 | 35.8700 |
|  | JLT2 | 0.0116 | 1.2700 | 0.2357 | 0.1236 | 90.4858 | 35.0500 |
|  | JLT1 | 0.0066 | 1.3702 | 0.2242 | 0.2688 | 92.3155 | 32.8900 |
|  | Mean | 0.0101 | 1.3947 | 0.2182 | 0.1978 | 93.0880 | 34.6033 |
| HTNE | HTNE3 | 0.0056 | 0.2961 | 0.0807 | 0.1401 | 67.6755 | 52.1900 |
|  | HTNE2 | 0.0052 | 0.1570 | 0.0743 | 0.0917 | 60.4787 | 48.5100 |
|  | HTNE1 | 0.0052 | 0.1513 | 0.0739 | 0.1195 | 70.8470 | 51.6300 |
|  | Mean | 0.0053 | 0.2014 | 0.0763 | 0.1171 | 66.3337 | 50.7767 |
| HSH | HSH3 | 0.0088 | 0.6373 | 0.1212 | 0.3927 | 68.7733 | 67.5100 |
|  | HSH2 | 0.0068 | 0.7497 | 0.1267 | 0.3711 | 90.9737 | 53.4400 |
|  | HSH1 | 0.0068 | 0.7874 | 0.1068 | 0.3078 | 79.2636 | 67.5100 |
|  | Mean | 0.0075 | 0.7248 | 0.1183 | 0.3572 | 79.6702 | 62.8200 |
| HJQ | HJQ3 | 0.0095 | 1.7988 | 0.1409 | 0.2317 | 87.1923 | 75.2600 |
|  | HJQ2 | 0.0116 | 1.5013 | 0.1999 | 0.2929 | 77.7999 | 77.7700 |
|  | HJQ1 | 0.0119 | 1.3853 | 0.1426 | 0.2311 | 83.0450 | 92.5700 |
|  | Mean | 0.0110 | 1.5618 | 0.1611 | 0.2519 | 82.6791 | 81.8667 |
| GEM | GEM3 | 0.0149 | 0.5232 | 0.0793 | 0.4604 | 65.3579 | 44.5700 |
|  | GEM2 | 0.0096 | 1.0392 | 0.0811 | 0.4028 | 57.7951 | 44.5700 |
|  | GEM1 | 0.0149 | 0.9581 | 0.1016 | 0.3318 | 61.6985 | 24.4500 |
|  | Mean | 0.0131 | 0.8401 | 0.0873 | 0.3983 | 61.6172 | 37.8633 |
| ETK | ETK3 | 0.0088 | 1.1675 | 0.1045 | 0.0532 | 72.6767 | 30.9800 |
|  | ETK2 | 0.0084 | 1.2640 | 0.1436 | 0.0786 | 71.5789 | 37.3400 |
|  | ETK1 | 0.0084 | 1.2221 | 0.1245 | 0.0664 | 70.6030 | 34.8300 |
|  | Mean | 0.0085 | 1.2178 | 0.1242 | 0.0660 | 71.6195 | 34.3833 |
| DFZ | DFZ3 | 0.0074 | 1.2879 | 0.0315 | 0.1356 | 51.0862 | 29.8100 |
|  | DFZ2 | 0.0062 | 1.6217 | 0.0368 | 0.1520 | 57.1852 | 24.6600 |
|  | DFZ1 | 0.0076 | 1.6228 | 0.0361 | 0.1596 | 51.0862 | 24.6600 |
|  | Mean | 0.0070 | 1.5108 | 0.0348 | 0.1490 | 53.1192 | 26.3767 |
| CSM | CSM3 | 0.0065 | 0.4958 | 0.1411 | 0.3676 | 63.5282 | 75.0800 |
|  | CSM2 | 0.0064 | 0.5849 | 0.1123 | 0.2851 | 57.0633 | 88.5400 |
|  | CSM1 | 0.0055 | 0.3174 | 0.1156 | 0.2940 | 64.3821 | 84.9500 |
|  | Mean | 0.0061 | 0.4660 | 0.1230 | 0.3156 | 61.6578 | 82.8567 |
| BMH | BMH3 | 0.0054 | 1.4924 | 0.1321 | 0.1526 | 60.9666 | 71.0600 |
|  | BMH2 | 0.0045 | 1.5708 | 0.1342 | 0.1141 | 60.7227 | 50.3700 |
|  | BMH1 | 0.0051 | 1.4976 | 0.1528 | 0.1148 | 53.7698 | 50.3700 |
|  | Mean | 0.0050 | 1.5203 | 0.1397 | 0.1272 | 58.4864 | 57.2667 |
| YG | YG3 | 0.0071 | 0.1104 | 0.0119 | 0.1953 | 28.2760 | 67.9000 |
|  | YG2 | 0.0072 | 0.1746 | 0.0116 | 0.1977 | 32.0574 | 69.5400 |
|  | YG1 | 0.0067 | 0.1679 | 0.0115 | 0.2053 | 32.4233 | 67.9400 |
|  | Mean | 0.0070 | 0.1510 | 0.0117 | 0.1994 | 30.9189 | 68.4600 |
| GH | GH3 | 0.0064 | 0.2337 | 0.0126 | 0.3063 | 33.5211 | 79.5900 |
|  | GH2 | 0.0065 | 0.1112 | 0.0133 | 0.2917 | 28.2760 | 106.1200 |
|  | GH1 | 0.0069 | 0.1075 | 0.0137 | 0.3051 | 34.6189 | 79.5900 |
|  | Mean | 0.0066 | 0.1508 | 0.0132 | 0.3010 | 32.1387 | 88.4333 |
| SNT | SNT3 | 0.0114 | 0.1442 | 0.0101 | 0.3665 | 48.1587 | 35.7900 |
|  | SNT2 | 0.0109 | 0.1160 | 0.0072 | 0.3043 | 45.8411 | 30.1200 |
|  | SNT1 | 0.0095 | 0.2215 | 0.0085 | 0.3263 | 46.9389 | 22.5400 |
|  | Mean | 0.0106 | 0.1606 | 0.0086 | 0.3323 | 46.9796 | 29.4833 |
| YQ | YQ3 | 0.0070 | 6.3144 | 0.5241 | 0.1150 | 101.7079 | 47.2100 |
|  | YQ2 | 0.0070 | 6.2506 | 0.4515 | 0.1087 | 112.1982 | 46.2600 |
|  | YQ1 | 0.0066 | 5.8148 | 0.5116 | 0.1127 | 108.5388 | 46.2600 |
|  | Mean | 0.0068 | 6.1266 | 0.4957 | 0.1121 | 107.4817 | 46.5767 |
| TGGLNE | TGGLNE3 | 0.0067 | 3.8147 | 0.3324 | 0.4119 | 128.0556 | 31.3700 |
|  | TGGLNE2 | 0.0066 | 2.5732 | 0.3777 | 0.4631 | 113.9059 | 21.8100 |
|  | TGGLNE1 | 0.0053 | 2.0396 | 0.3744 | 0.4354 | 139.1558 | 28.1300 |
|  | Mean | 0.0062 | 2.8092 | 0.3615 | 0.4368 | 127.0391 | 27.1033 |
| SHT | SHT3 | 0.0061 | 2.0631 | 0.0765 | 0.3676 | 122.8105 | 43.5800 |
|  | SHT2 | 0.0059 | 3.6231 | 0.0992 | 0.1912 | 112.1982 | 31.1100 |
|  | SHT1 | 0.0064 | 3.6422 | 0.1167 | 0.2940 | 136.8382 | 40.9800 |
|  | Mean | 0.0062 | 3.1095 | 0.0975 | 0.2843 | 123.9490 | 38.5567 |
| DLT | DLT3 | 0.0165 | 3.6856 | 0.4815 | 0.4408 | 90.7297 | 43.4500 |
|  | DLT2 | 0.0121 | 2.9307 | 0.4827 | 0.4630 | 97.6826 | 33.6700 |
|  | DLT1 | 0.0123 | 2.9048 | 0.4576 | 0.4583 | 100.1222 | 22.9800 |
|  | Mean | 0.0136 | 3.1737 | 0.4740 | 0.4540 | 96.1782 | 33.3667 |
| BDJL | BDJL3 | 0.0096 | 2.3667 | 0.2190 | 0.4517 | 111.7103 | 46.2200 |
|  | BDJL2 | 0.0097 | 2.9212 | 0.2957 | 0.4393 | 149.8900 | 31.9300 |
|  | BDJL1 | 0.0087 | 2.9212 | 0.1888 | 0.4820 | 130.0073 | 25.5300 |
|  | Mean | 0.0093 | 2.7364 | 0.2345 | 0.4576 | 130.5359 | 34.5600 |

**Table S7.** ANOVA analysis of bioactive components in *C. songaricum.*

|  | | Sum of Squares | df | Mean Square | F | Sig. |
| --- | --- | --- | --- | --- | --- | --- |
| 3,4-dihydroxybenzaldehyde | Between Groups | 0.001 | 27 | 0.000 | 16.306 | < 0.01 |
|  | Within Groups | 0.000 | 56 | 0.000 |  |  |
|  | Total | 0.001 | 83 |  |  |  |
| Catechin | Between Groups | 134.554 | 27 | 4.983 | 44.131 | < 0.01 |
|  | Within Groups | 6.324 | 56 | 0.113 |  |  |
|  | Total | 140.877 | 83 |  |  |  |
| Epicatechin | Between Groups | 1.294 | 27 | 0.048 | 118.421 | < 0.01 |
|  | Within Groups | 0.023 | 56 | 0.000 |  |  |
|  | Total | 1.317 | 83 |  |  |  |
| Ursolic acid | Between Groups | 1.292 | 27 | 0.048 | 34.051 | < 0.01 |
|  | Within Groups | 0.079 | 56 | 0.001 |  |  |
|  | Total | 1.371 | 83 |  |  |  |
| ‎ Total phenolics | Between Groups | 63644.637 | 27 | 2357.209 | 51.430 | < 0.01 |
|  | Within Groups | 2566.658 | 56 | 45.833 |  |  |
|  | Total | 66211.295 | 83 |  |  |  |
| Crude polysaccharides | Between Groups | 45867.760 | 27 | 1698.806 | 26.531 | < 0.01 |
|  | Within Groups | 3585.690 | 56 | 64.030 |  |  |
|  | Total | 49453.450 | 83 |  |  |  |

# Supplementary Equations

#### ****Partial Least Squares Regression Equations for Bioactive Components in**** Cynomorium songaricum

The Partial Least Squares Regression (PLSR) revealed the relationship between environmental factors and six chemical components of C. songaricum. The resulting equations are presented below:

**Equation S1.** Regression equation for 3,4-dihydroxybenzaldehyde (Y₁):
Y₁ = 0.007 + 0.004×X₅ − 0.000×X₂ − 0.002×X₃ − 0.002×X₇ − 0.001×X₉ + 0.002×X₈ + 0.001×X₄ + 0.000×X₆ + 0.002×X₁ + 0.004×X₁₀ − 0.001×X₁₁ − 0.001×X₁₂ − 0.000×X₁₃ + 0.001×X₁₄ − 0.002×X₁₅ − 0.001×X₁₆ − 0.003×X₁₇ + 0.000×X₁₈
(R² = 0.601)

**Equation S2.** Regression equation for catechin (Y₂):
Y₂ = 1.408 + 0.774×X₅ − 0.049×X₂ + 0.129×X₃ − 0.577×X₇ − 0.571×X₉ + 0.753×X₈ − 0.144×X₄ − 0.477×X₆ + 0.236×X₁ + 0.895×X₁₀ + 0.055×X₁₁ − 0.077×X₁₂ − 0.196×X₁₃ + 0.325×X₁₄ − 0.128×X₁₅ + 0.017×X₁₆ − 0.281×X₁₇ + 0.142×X₁₈
(R² = 0.541)

**Equation S3.** Regression equation for epicatechin (Y₃):
Y₃ = 0.144 + 0.049×X₅ + 0.008×X₂ + 0.023×X₃ − 0.022×X₇ − 0.089×X₉ + 0.053×X₈ − 0.009×X₄ − 0.020×X₆ + 0.024×X₁ + 0.109×X₁₀ − 0.056×X₁₁ − 0.002×X₁₂ − 0.067×X₁₃ − 0.033×X₁₄ − 0.017×X₁₅ + 0.000×X₁₆ − 0.080×X₁₇ − 0.036×X₁₈
(R² = 0.530)

**Equation S4.** Regression equation for ursolic acid (Y₄):
Y₄ = 0.228 + 0.074×X₅ + 0.010×X₂ − 0.044×X₃ − 0.015×X₇ + 0.040×X₉ + 0.007×X₈ + 0.042×X₄ − 0.056×X₆ + 0.035×X₁ + 0.057×X₁₀ − 0.064×X₁₁ + 0.006×X₁₂ − 0.047×X₁₃ + 0.023×X₁₄ − 0.065×X₁₅ + 0.127×X₁₆ + 0.003×X₁₇ + 0.021×X₁₈
(R² = 0.503)

**Equation S5.** Regression equation for total phenolic content (Y₅):
Y₅ = 75.256 + 11.884×X₅ + 0.794×X₂ + 3.904×X₃ − 10.445×X₇ + 1.412×X₉ + 9.970×X₈ − 2.105×X₄ − 20.405×X₆ + 2.937×X₁ + 5.125×X₁₀ − 8.771×X₁₁ + 18.125×X₁₂ − 6.860×X₁₃ − 5.373×X₁₄ + 5.488×X₁₅ + 5.024×X₁₆ + 6.232×X₁₇ − 8.651×X₁₈
(R² = 0.525)

**Equation S6.** Regression equation for crude polysaccharide fraction (Y₆):
Y₆ = 54.083 − 28.754×X₅ − 1.786×X₂ + 4.498×X₃ + 16.710×X₇ + 3.934×X₉ − 3.002×X₈ − 5.099×X₄ + 26.621×X₆ + 2.585×X₁ − 3.740×X₁₀ + 0.881×X₁₁ − 0.146×X₁₂ − 0.366×X₁₃ − 14.336×X₁₄ + 8.859×X₁₅ − 10.709×X₁₆ + 0.168×X₁₇ + 5.245×X₁₈
(R² = 0.603)

**Variable Definitions:**

- **Y₁–Y₆** represent concentrations of:
  - Y₁: 3,4-dihydroxybenzaldehyde
  - Y₂: catechin
  - Y₃: epicatechin
  - Y₄: ursolic acid
  - Y₅: total phenolic content
  - Y₆: crude polysaccharide fraction
- **X₁–X₁₈** represent normalized environmental variables:
  X₁: BIO3 – Isothermality
  X₂: BIO5 – Max temperature of warmest month
  X₃: BIO6 – Min temperature of coldest month
  X₄: BIO7 – Temperature annual range
  X₅: BIO13 – Precipitation of wettest month
  X₆: BIO15 – Precipitation seasonality
  X₇: BIO19 – Precipitation of coldest quarter
  X₈: hsdgs – Sunshine duration in growing season
  X₉: BIO2 – Mean diurnal range
  X₁₀: tpd – Total phosphorus density in soil
  X₁₁: tn – Total nitrogen in soil
  X₁₂: slope – Slope gradient
  X₁₃: pH (30–60 cm)
  X₁₄: cf – Coarse fragment
  X₁₅: cec – Cation exchange capacity of soil
  X₁₆: btslt – Soil silt content
  X₁₇: bd – Bulk density of soil
  X₁₈: aspect
